# Algorithms for Scanned Probe Microscope Image Simulation, Surface Reconstruction, and Tip Estimation

**DOI:** 10.6028/jres.102.030

**Published:** 1997

**Authors:** J. S. Villarrubia

**Affiliations:** National Institute of Standards and Technology, Gaithersburg, MD 20899-0001

**Keywords:** algorithms, atomic force microscopy, blind reconstruction, dimensional metrology, image simulation, mathematical morphology, scanned probe microscopy, scanning tunneling microscopy, surface reconstruction, tip artifacts, tip estimation

## Abstract

To the extent that tips are not perfectly sharp, images produced by scanned probe microscopies (SPM) such as atomic force microscopy and scanning tunneling microscopy are only approximations of the specimen surface. Tip-induced distortions are significant whenever the specimen contains features with aspect ratios comparable to the tip’s. Treatment of the tip-surface interaction as a simple geometrical exclusion allows calculation of many quantities important for SPM dimensional metrology. Algorithms for many of these are provided here, including the following: (1) calculating an image given a specimen and a tip (dilation), (2) reconstructing the specimen surface given its image and the tip (erosion), (3) reconstructing the tip shape from the image of a known “tip characterizer” (erosion again), and (4) estimating the tip shape from an image of an unknown tip characterizer (blind reconstruction). Blind reconstruction, previously demonstrated only for simulated noiseless images, is here extended to images with noise or other experimental artifacts. The main body of the paper serves as a programmer’s and user’s guide. It includes theoretical background for all of the algorithms, detailed discussion of some algorithmic problems of interest to programmers, and practical recommendations for users.

## 1. Introduction

Accurate length metrology of sub-micrometer surface features is important for a variety of technologies. Determination of grain size [1] or surface microroughness [2] and comparison of measured dimensions of organic molecules to calculated models [3] all require accuracy on the scale of nanometers or better. The Semiconductor Industry Association has identified critical dimension metrology at this scale as an important item on the path to the next generation of semiconductor electronics [4].

Scanned probe microscopy (SPM), chiefly scanning tunneling microscopy (STM) and atomic force microscopy (AFM) are promising newcomers as length metrology tools. They provide three-dimensional images with resolution at or near the atomic level. However, while a high resolution image is an important requisite for accurate measurement of dimensions, it is not sufficient. One problem, geometrical distortion in the images due to nonlinearities in the scanners, can be overcome through the use of interferometry or calibrated capacitance gauges to traceably measure the position of the tip relative to the sample [5, 6]. Another impediment is image distortion due to dilation of image features by the tip. Overcoming this obstacle requires methods of estimating the tip geometry and using the estimate to reconstruct the true specimen shape from its measured image. Perhaps the earliest proposed solution to this problem was that of Reiss et al. [7]. Keller provided an alternative formulation in terms of Legendre transforms [8]. Other authors [9–11] have published solutions which are essentially specializations of these to particular geometries, e.g., spheres or parabolas. These solutions rely upon the principle that non-interpenetrating surfaces in contact must be tangent at the contact point. For reconstructing general tip shapes, they require numerical evaluation of slopes. This has sometimes been found problematical in practice [12]. As a result another approach relying upon mathematical morphology has been used by a number of authors [12–18]. These are applicable to general shapes (any tip and sample which can be expressed as an array of heights in the usual fashion), and they do not require numerical derivatives.

Any of these methods can be used to reconstruct from an image either the specimen surface if the tip is known or the tip geometry if the specimen is known. For tip estimation, the requirement that the tip characterizer specimen be known independently of the SPM measurement can be a significant hurdle. This kind of three dimensional nanometer resolution measurement of the characterizer is not easily performed by non-SPM techniques. Even if it is once known, one must still be concerned with the stability of the characterizer and registry. That is, how does one know that the specimen, once accurately measured, does not change due to contamination, reaction, or other damage, and how does one know whether the area being imaged in the SPM is the same area previously measured? This author recently published an alternative to tip estimation using known tip characterizers [18]. Williams et al. [19] independently arrived at essentially identical conclusions. Dongmo et al. [20] describe a different, but related, technique. These methods, which have come to be known as “blind reconstruction” methods, determine an outer bound on the tip geometry from an image of an object without a priori knowledge of the object’s actual geometry. For well-chosen tip characterizers, the outer bound determined by these methods may closely approximate the actual tip geometry [21].

The primary purpose of this paper is to make the results derived in Ref. [18] available in a practically implementable form. To that end, actual computer codes (in C) for image simulation, surface reconstruction, tip estimation, and related operations are provided in the appendices. A secondary purpose is to extend the previous results in an important respect. The original blind reconstruction algorithm, while useful for modeling, had practical problems with real images due to an instability in the presence of noise. The code presented here employs a threshold test to remove the instability.

There are three main tasks accomplished in the body of the paper. Firstly, a reasonably comprehensive theoretical basis for the algorithms is established, though the derivation of blind reconstruction is omitted since this is long and was already published in Ref. [18]. The theoretical basis is necessary so that users may understand what the algorithms calculate, understand the principles upon which they are based, and judge the reasonableness of results they generate. Secondly, some details of how the mathematical results are embodied in algorithms are given, especially when it would not otherwise be obvious. As a practical matter, for example, images are measured only over a finite area. Sometimes the formulas as derived require information from parts of the image that lie beyond the edge, in unknown territory. The solution to this problem will be given. Thirdly, some practical guidance to users will be offered.

The organization of the paper is as follows: In Sec. 2 the basic mathematical concepts and notation will be introduced. Section 3 is on image simulation (calculating the image given the specimen and tip). Section 4 is on surface reconstruction and certainty maps (estimating the specimen given the image and the tip or the tip given the image and specimen, and determining where the reconstruction is valid). Section 5 covers blind reconstruction (estimating the tip shape using the image alone). Each of Secs. 3 through 5 derives or recapitulates the relevant equations, then discusses how these are implemented in algorithms. In Sec. 6 we discuss the effect of noise and other limitations. Section 7 provides some practical examples. The appendices contain computer code for practical implementation of the algorithms described in the main body of the paper.

## 2. The Language of Sets

Mathematical morphology, a branch of set theory dealing with unions and intersections of sets and their translates, provides a useful language for problems related to SPM. For this reason basic routines for the morphological operations of dilation and erosion are provided in Appendix C. As we will see in Sec. 3, imaging can be compactly described in terms of dilation. Once the connection between SPM and dilation is established, the existence of mathematical morphology as a branch of set theory means there exist proven relationships between morphological operations which may be usefully applied to SPM. For example, in Sec. 4 there is a brief, straightforward proof that the erosion operation produces the best obtainable surface reconstruction. Neither grayscale morphology, a subset of mathematical morphology to which we will shortly restrict ourselves, nor a surface description of objects based upon single-valued functions (the more conventional approach) can describe surfaces or tips with undercuts. However it is worth mentioning that mathematical morphology, when not restricted to grayscale morphology, is applicable to such surfaces.

We will introduce definitions and properties of morphological operators as we need them. Motivation for the former and proofs of the latter may be found in the morphology literature [22–26]. However, since it may be unfamiliar, we introduce some of the notation and basic ideas here. In most treatments of SPM imaging, the image, specimen, and tip surfaces are described in terms of single-valued functions which give the height of the corresponding object at the given lateral coordinates, (*x*, *y*). Thus, *s*(*x*, *y*) is the upper surface (the “top”) of the specimen. In mathematical morphology, the specimen is described by the set, *S*, of all the points contained within the specimen volume. When only the upper surface of *S* is relevant, as in standard SPM imaging, we can treat *S* as though it were defined by *S* = {(*x*, *y*, *z*)|*z* ≤ *s*(*x*, *y*)}. This kind of an object, which consists of a single-valued top and all the points beneath it, is called an “umbra.” The transformation between a description in terms of an umbra on the one hand and its top on the other provides the translation between mathematical morphology and the standard description.

The standard description is a boundary representation, while mathematical morphology represents objects as solids occupying a volume. Each has its advantages and disadvantages. The volume description comes with a compact and intuitive notation, as we will shortly see. It also has the virtue of being an established body of study, with definitions and theorems useful to our purpose. The boundary description, on the other hand, is arguably sufficient for SPM. Tips and specimens interact at their surfaces. When we know what the solid object’s boundaries are, we have all we need. To perform calculations on the objects’ interior points would be inefficient. It is often convenient to take advantage of the existing notation and theorems of mathematical morphology to perform derivations, but convert the results to surface descriptions for computational efficiency when it comes time to encode them.

Since a facility for going back and forth between the alternate descriptions will be useful to us, here are a few examples of important operations expressed both ways. The translation of a set, *A*, by a vector, ***d***, is determined by adding ***d*** to every element of *A*:

A+d={a+d|a∈A} (Definition 1)

**Fig. 1:**
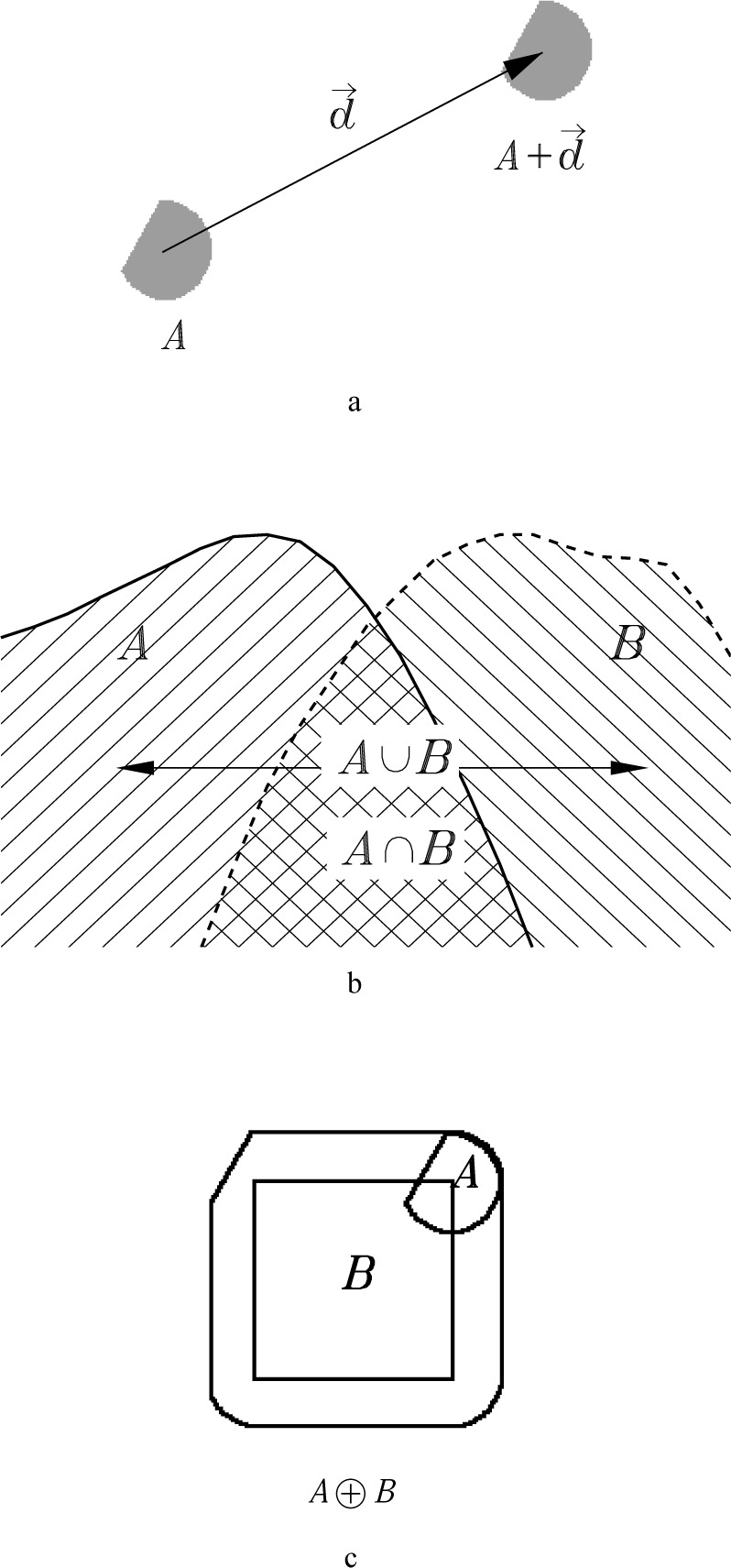
Some basic operations on sets. a) Translation of a set by a vector. b) Union and intersection of sets, and their relationship to the maximum and minimum of the tops of the sets. c) Dilation of one set by another.

This is shown graphically in Fig. 1a. If A were an umbra, the corresponding description of the translation in terms of its top would be *a*(*x*−*d_x_*, *y*−*d_y_*) + *d_z_*, where ***d*** = (*d_x_*, *d_y_*, *d_z_*). That is, denoting the top of *A* by *T* [*A*],

$$T\left[A+d\right]\left(x,y\right)=a\left(x-d_{x},y-d_{y}\right)+d_{z}.$$ (Property 1)

Two overlapping umbras are shown in Fig. 1b. The union of the two umbras is represented by all of the shaded area, regardless of the orientation of the shading lines. It is clear from the definitions that the top of the union is the maximum of the two tops.

T[A∪B](x,y)=max[a(x,y),b(x,y)]. (Property 2)

Similarly, the intersection is the area of the figure which is shaded by both umbras. It is the crosshatched area, and its top is the minimum of the two tops.

T[A∩B](x,y)=min[a(x,y),b(x,y)]. (Property 3)

For our final example, which we will use shortly, we introduce the definition of dilation.

$$A⊕B=\underset{b\in B}{\cup }\left(A+b\right)$$ (Definition 2)

This definition as a union of translates is illustrated in Fig. 1c. Here we take the point *a* = 0 to be at the center of curvature for the curved part of *A*. The position of *A* in the figure shows one of the translates, *A* + ***b***. In this instance, ***b*** is the point at the upper right corner of *B*. If one imagines centering *A* in turn over each ***b*** in *B*, the area swept out by *A* is the dilation, labelled *A* ⊕ *B* in the figure. *A* and *B* were chosen not to be umbras in order to illustrate the generality of the definition. However, if we do restrict consideration to umbras, we can use Definition 1 and Property 2 to write an expression for the function defining the top of the dilation

$$T\left[A⊕B\right]\left(x,y\right)=\underset{\left(u,v\right)}{\max}\left[a\left(x-u,y-v\right)+b\left(u,v\right)\right].$$ (Property 4)

## 3. Simulation of Imaging, Dilation

### 3.1 A Model for Imaging

**Fig. 2:**
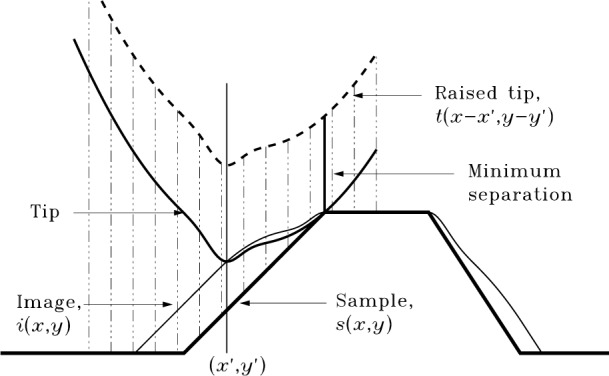
The conventional model for imaging.

Figure 2 illustrates the principles of AFM or STM topographic imaging. This and most subsequent figures show only profiles for the sake of clarity, but the results and the algorithms in the appendices are applicable to full three dimensional surfaces. A tip is positioned above the specimen surface. The tip then approaches the surface until it makes contact at one or more points. When it makes contact, the location of the tip apex defines the image height. The practical meaning of “contact,” and the degree of approximation implicit in this model, are determined by the feedback mechanism employed. In the STM for example, feedback is based on the tunneling current between a conducting tip and surface between which a potential difference is maintained. The tunneling gap is typically less than 1 nm. In constant current imaging, the gap should remain constant apart from variations on the order of tenths of a nanometer due to work function variations. The amount of compression for hard samples and tips in contact mode AFM at typical forces should be of similar order. Therefore, the approximation of contact without compression should be valid at the size scales large compared to 1 nm which are of interest for much of the topography of patterned semiconductors, microcrystals, and other surfaces.

### 3.2. The Imaging Equation

What is a mathematical description of the process just described? In Fig. 2 let the coordinate system be chosen so the height of the apex of the raised tip is 0. Let *i* (*x*, *y*) be the function describing the image surface, *s*(*x*, *y*) the specimen, and *t*(*x*, *y*) the tip. When the tip is translated to the point (*x*′, *y*′) the translated tip is described by *t*(*x*−*x*′, *y*−*y*′). The tip must be lowered until it first touches the surface. That is, it must be translated down by an amount equal to the minimum distance between tip and specimen surface. Representative distances as a function of lateral position are shown by the dashed vertical lines, with the minimum distance indicated by the thick continuous line at the upper corner of the sample. When the tip is lowered into contact, the apex will mark the height of the image at (*x*′, *y*′). That is,

$$i\left(x^{\prime },y^{\prime }\right)=-\underset{\left(x,y\right)}{\min}\left[t\left(x-x^{\prime },y-y^{\prime }\right)-s\left(x,y\right)\right].$$ (1)

Here the minimum is taken over all (*x*, *y*) in the horizontal plane.

### 3.3 Equivalence of Imaging to Dilation

Now we make a few algebraic manipulations, the point of which is to demonstrate the relationship between Eq. (1) and dilation. First, we bring the leading minus sign inside the *min* operation, thereby converting it to a *max* operation, since − *min* (*a*) = *max* (−*a*). At this point the result agrees, apart from notational differences, with imaging equations in Refs. [12], [14], and [15]. Second, we introduce a change of variables, *x* = *x*′ − *u* and *y* = *y*′ − *v*. With these changes, Eq. (1) becomes

$$i\left(x^{\prime },y^{\prime }\right)=\underset{\left(u,v\right)}{\max}\left[s\left(x^{\prime }-u,\;y^{\prime }-v\right)-t\left(-u,-v\right)\right].$$ (2)

Here we have used the fact that $\underset{x^{\prime }-u}{\max}=\underset{u}{\max}$. (Since *u* varies from −∞ to + ∞, *x*′−*u* and *u* represent the same region, only specified in a different order.) Now define a new function,

p(x,y)=−t(−x,−y), (3)

which is the reflection of the tip through the origin. In terms of this new function, Eq. (2) becomes

$$i\left(x,y\right)=\underset{\left(u,v\right)}{\max}\left[s\left(x-u,y-v\right)+p\left(u,v\right)\right].$$ (4)

By comparing with Property 4, it is apparent that in agreement with others [13, 14, 17], Eq. (4) means

**Fig. 3:**
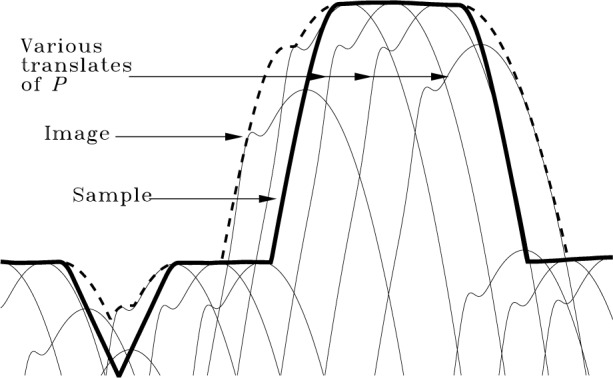
Forming the image by dilation.

I=S⊕P (5)

where *I*, *S*, and *P* are the sets of which the functions *i*, *s*, and *p* are the respective tops. That imaging is, in fact, a dilation is further illustrated in Fig. 3. This figure shows the same geometrical operation demonstrated in Fig. 1 when we defined dilation, but using the sample (thick line) and reflection of the tip introduced at Fig. 2. The coordinate system is assumed chosen so that the apex of the untranslated tip lies at the origin. Some of the various translates of the reflected tip are shown in the figure. The image (dashed line) produced by dilation in Fig. 3 is the same image determined by the more conventional operation described in Fig. 2.

### 3.4 Algorithms for Reflection and Dilation

In order to simulate imaging using the codes in the appendices, it is necessary to have tip and model surfaces expressed as two dimensional arrays of heights. Such height maps are the standard way in which SPM images are stored. To process 1-d data (profiles of height vs *x*) one simply uses arrays which are formally 2-d but with one of the dimensions having size equal to 1. The algorithms provided operate on integer arrays specified by pointers of type long **. A utility to allocate arrays of this type is supplied in Sec. 10.2. Generalization to data types other than long integer is straightforward. (See the discussion in Appendix A.)

In Sec. 10.3 the ireflect routine performs the reflection operation, *P* = − *T*, useful if the tip is not already in reflected form. The algorithm is a short and straightforward implementation of the definition of reflection through the origin. The order of the height values within the array is reversed in each of the *x* and *y* directions, and the sign of the result is changed to produce the inversion in *z*.

The idilation routine in Sec. 11.1 is a mostly straightforward implementation of dilation as given in Eq. 4. As inputs, it requires pointers to arrays containing the height data for the sample surface and the reflected tip and the dimensions of these two arrays.

There is a small complication in the implementation of Eq. (4) which arises over the choice of coordinate system. Since we represent *s* and *p* by arrays, it is convenient to use the integer index into the arrays as the lateral coordinates, *x*, *y*, *u*, and *v* in Eq. (4). This represents no complication with regard to image or specimen arrays, but does raise a problem for the tip array. On the one hand it is convenient and natural to place the tip apex at the origin, as we did in deriving Eq. (4). Other choices result in the image being translated with respect to the specimen. On the other hand, arrays in C are naturally zero-offset, with (0, 0) in the lower left corner. This is not usually a good place to put the tip apex, since the array then describes only a single quadrant of the tip. The solution is to make the natural choice of tip array, with the apex at some (*x*_c_, *y*_c_) in the interior, and then index the array with (*u* + *x*_c_, *v* + *y*_c_) instead of (*u v*). Now *u* and *v* can range more or less symmetrically about 0, as we want them to in Eq. (4), while the array index remains in the appropriate range for the programming language. The procedure just described amounts to generating a new function, *p*_c_(*u* + *x*_c_, *v* + *y*_c_), which is equal to and replaces in *p*(*u*, *v*) in Eq. (4). In the idilation routine and others to follow, the tip variable refers to *p*_c_. This explains the difference between line 77 in the code and what one might expect by inspection of Eq. (4).

The outermost pair of loops, beginning at lines 68 and 71, ranges in turn over each (*x*, *y*) in the image. For each such (*x*, *y*) coordinate, the inner loops, beginning at lines 75 and 76, range over all (*u* + *x*_c_, *y* + *y*_c_) in the domain of the tip, computing for each the value of the expression in Eq. (4)’s square brackets and finally determining the maximum of these.

Another complication which rears its head here for the first, but not last, time is the existence of edges. In our discussion of the last section we assumed the image, specimen, and tip were described by functions defined for all (*x*, *y*) in the horizontal plane. In fact, however, we are always given only truncated representations of these objects. Among the many different translates of the tip are some in which part of the tip lies over the edge of the known specimen surface. In this situation, two issues must be addressed.

First, we must use care in coding in order not to attempt to address parts of *s* or *p*_c_ outside the specified arrays. For a given *x* the conditions on the range of *u* are

$$\begin{array}{l} 0\leq x-u\leq \text{surf\_xsiz}-1\\ 0\leq u+x_{c}\leq \text{tip\_xsiz}-1. \end{array}$$ (6)

The first of these comes from requiring the argument of *s* in Eq. (4) to be within the defined domain of *s*. The second comes from the similar requirement on the argument of *p*_c_. To satisfy both the conditions of Eq. (6), it is necessary that

$$\begin{array}{l} \;\max\left[x-\text{surf\_xsiz}+1,-x_{c}\right]\\ \leq u\leq \min\left[\text{tip\_xsiz}-x_{c}-1,x\right]. \end{array}$$ (7)

A similar condition applies to *v*, and the conditions are applied in lines 69, 70, 72, and 73.

Second, we must decide what value should be assigned to the *max* operation of Eq. (4) when its range includes parts of the specimen surface for which we have no data. In the case of dilation, we here assume that heights of the reflected tip or specimen surface which are not otherwise defined may be taken to be − ∞. Algorithmically, this means that those areas may be ignored when determining the maximum. Physically, this means we are assuming that we are provided with all parts of the tip and specimen that are relevant to the image.

## 4. Reconstruction of Surfaces, Erosion and Certainty Maps

### 4.1 The Reconstruction Equation

A common problem is, given a measured image and an estimate for the tip shape, how do we estimate the specimen surface? The answer is

$$S_{r}=I⊖P.$$ (8)

The ⊖ symbol designates erosion, defined by

$$A⊖B=\underset{b\in B}{\cap }\left(A-b\right).$$ (Definition 3)

We will see that *S*_r_ is an upper bound, and not necessarily equal to *S*. On the other hand, *S*_r_ is not only a reconstruction of the surface, but it is, within the model given in the last section, the *best possible* reconstruction. Other reconstruction procedures which start with the same model are either equivalent to *S*_r_, or worse than *S*_r_. In fact, there have been a number of reconstruction procedures [7–18]. Although few are stated explicitly in terms of morphological operators most appear to be formally equivalent, although some require a problematical evaluation of numerical derivatives or are restricted to certain tip geometries. To see that *S*_r_ is the best possible reconstruction, we need two properties from mathematical morphology.

(A⊕B)⊖B⊇A. (Property 5)

[(A⊕B)⊖B]⊕B=A⊕B, (Property 6)

Since *I* = *S* ⊕ *P*, Property 5 and Eq. (8) say that

$$S_{r}⊇S.$$ (9)

This means *S*_r_ contains, or is an upper bound on, the actual surface. That it is the *least* such upper bound consistent with the image may be seen using Property 6, which upon substitution of *S* for *A*, *P* for *B*, *I* for *S* ⊕ *P* and *S*_r_ for *I* ⊖ *P* says that

$$S_{r}⊕P=I.$$ (10)

This means that if the specimen were equal to *S*_r_, we would have produced precisely the observed image. It is therefore not possible to eliminate *S* = *S*_r_ as a possibility. As a result, no upper bound smaller than *S*_r_ is acceptable, and *S*_r_ is the *least* upper bound.

**Fig. 4:**
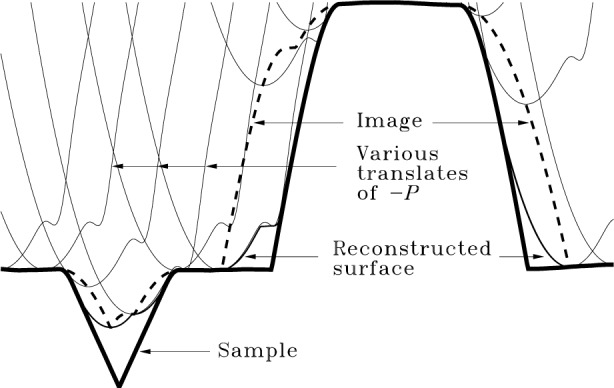
Geometrical interpretation of erosion, showing that it is the surface of deepest penetration. The specimen surface is the thick continuous line. The image is the dashed line. Various translates of the tip are shown, together with the minimum of their envelope, which is the reconstructed surface.

A geometrical picture of erosion is presented in Fig. 4 with the aid of yet another result from mathematical morphology:

$$I⊖P={\left[I^{c}⊕\left(-P\right)\right]}^{c}.$$ (Property 7)

This shows that erosion is related to (is, in fact, the dual of) dilation. Here *X*^c^ denotes the complement of *X*. In Fig. 4 *I* is the space below and including the image surface (dashed line). *I*^c^ is the space above the image. The dilation of *I*^c^ by −*P* is graphically constructed in similar fashion to that used in Fig. 3. The resulting object’s lower surface is indicated by the thin continuous line. The final complement operation performs another inversion, making this the *upper* surface of the result, *S*_r_. This graphical procedure is the same as that employed by Keller and Franke [12] under the name “envelope reconstruction,” which is therefore equivalent to erosion. The result is compared to the actual surface, shown by the thick continuous line.

Figure 4 provides a physical interpretation of surface reconstruction by erosion. *S*_r_ is an upper bound on *S* rather than equal to *S* because there are regions like those in the v-groove or near the base of steep walls which the tip is too large to penetrate. Altering *S* in these inaccessible regions makes no change in the image, and it is therefore not possible from the image alone to tell which of the many possibilities was the true one. *S_r_ is the best reconstruction because it is the surface of deepest penetration of the tip.*

If the specimen geometry is known but the tip is not, it is possible to use erosion to reconstruct the tip shape.

$$P_{r}=I⊖S.$$ (11)

In this case *I* is the image of the known reference specimen, *S*. Analogously to *S*_r_ and *S* in the foregoing discussion, *P*_r_ is an outer bound on the probe shape, equal to *P* at those points where *P* touched *S* and an outer bound elsewhere.

### 4.2 Erosion Algorithm

To put erosion (Definition 3) into a form suitable for programming, it is useful to have an expression for the top of *S*_r_. To this end we apply Property 1 (dealing with translations) and Property 3 (dealing with intersection) to Definition 3. The result is

$$\begin{array}{l} s_{r}\left(x,y\right)=T\left[S_{r}\right]\left(x,y\right)\\ =\underset{\left(u,v\right)}{\min}\left[i\left(x+u,y+v\right)-p\left(u,v\right)\right]. \end{array}$$ (12)

Section 11.2 contains the function, ierosion, which implements this equation for integer arrays. The inputs are pointers (of type long **) to arrays containing the image and tip, the sizes of these arrays, and coordinates within the tip which are to be considered the origin.

As with dilation, there are two sets of loops, an outer set for (*x*, *y*) and an inner one for (*u*, *v*). Line 104 evaluates the expression in Eq. (12)’s square brackets, with *p* offset as before by (*x*_c_, *y*_c_). (See the discussion in 3.4.) The inner set of loops determines the minimum over all (*u*, *v*) for a given (*x*, *y*).

As before, we must be careful about edges. The expressions in lines 96, 97, 99, and 100 were derived analogously to those for dilation, differing only because of the sign differences between the arguments of *i* in Eq. (12) and *s* in Eq. (4). These lines prevent us from attempting to address the image or tip arrays outside of their defined limits as we would otherwise attempt to do for those configurations in which part of the tip lies over the edge of the image.

As it stands, the *min* operation now proceeds only over those coordinates where both tip and image are defined. However, we still must consider whether this is the right thing to do, or whether some other value should be assigned to *min* when its range includes undefined regions of the image. The image is ordinarily a measured quantity, and we have no way of knowing what we would have measured had we extended the imaging region beyond its current boundaries. However, the spirit of this calculation is defined by the fact that *S*_r_ ⊇ *S*. We are calculating an *upper bound* on the actual specimen surface. In order to preserve this character to the calculation, we make the worst-case assumption, that is, we assume that value of *i* which maximizes the result for *s*_r_. In this way we guarantee that *s*_r_ is, in fact, an upper bound no matter what the true value of *i* beyond the edge. The assumption for *i* which maximizes *s*_r_ is that *i* → ∞ where it is not otherwise known. Algorithmically, this also means the unknown parts of *i* are irrelevant to the *min* procedure, and ierosion is correct as it stands.

### 4.3 Certainty Map

We have seen that it is not always possible to reconstruct the specimen surface from its image. In general, the reconstruction is equal to the specimen in some places and greater in others. Interestingly, it is sometimes possible to ascertain where the reconstruction worked. Pingali and Jain [14] suggested a procedure for constructing a “certainty map.” The certainty map, *c*(*x*, *y*), is an array of the same size as the reconstructed surface, but containing 1’s and 0’s. If *c*(*x*, *y*) = 1 for some pixel, (*x*, *y*), then *s*_r_(*x*, *y*) = *s*(*x*, *y*). Where *c*(*x*,*y*) = 0 the corresponding reconstructed pixel may or may not be equal to the true surface.

**Fig. 5:**
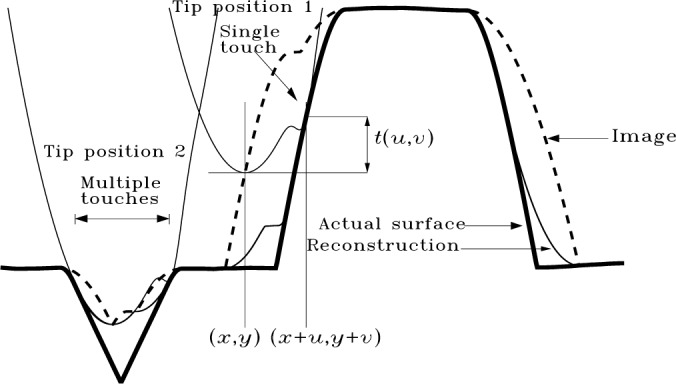
Two possible scenarios. The tip at position 1 touches the reconstructed surface (and therefore also the actual surface) at a single point. At position 2, the tip touches the reconstructed surface at multiple points, and it is not therefore possible to know which of them corresponds to the true surface.

Figure 5 shows how it works and why. The image is formed when the tip scans the surface, always in contact at one or more points. Two tip positions are shown in the figure. At position 1 the tip makes contact with *s*_r_ at one point. By process of elimination, this point is the *only* candidate for the place where the tip contacted the specimen. All other points are eliminated because *s*_r_ is known to be an upper bound on *s*. Therefore, *s* = *s*_r_ at this point. At position 2 the tip contacts the reconstructed surface at multiple points. At least one of these must coincide with the true surface, but it is not possible to say which.

### 4.4 Certainty Map Algorithm

An algorithm, icmap, to calculate the certainty map is given in Appendix D. It takes as inputs pointers to an image, a reflected tip (with center coordinates also given) and a reconstructed surface previously determined from these using ierosion. The main result which we need in order to convert the description of the last section to an algorithm is the condition under with the tip touches a point on the reconstructed surface. By inspection of Fig. 5 (see the labels at tip position 1) the tip at (*x*, *y*) touches the reconstructed surface at (*x* + *u*, *y* + *v*) if and only if

$$i\left(x,y\right)+t\left(u,v\right)=s_{r}\left(x+u,y+v\right).$$ (13)

This is the comparison which is performed at line 145. However, since Eq. (13) requires the unreflected tip and since we have standardized the algorithms on accepting the reflected ones as input, we must either call the ireflect routine or perform a reflection in place. The latter option is employed here. The innermost pair of loops (starting at lines 143 and 144) ranges over all (*u*, *v*) in the domain of the functions. The outer pair of loops ranges over all (*x*, *y*) not too near the edge. If Eq. (13) is true, the block following line 145 increments a counter which tallies the number of values of (*u*, *v*) for which there is a touch, and stores the location of the touch. If, at the end of each loop over all (*u*, *v*) there has been only a single touch, the certainty map at the stored location is set to 1.

As with the previously considered routines, it is necessary to consider the effect of edges. We will consider an image pixel to be near the edge of the image if, when the unreflected tip is placed with its center coordinates over that pixel, part of the tip lies over the edge. As written, icmap assigns a value of 0 to *all* such pixels since there may be additional touch points “unseen” beyond the edge.

As it happens, this is too conservative. It is possible to do better than this if we consider that specimen heights beyond the edge are not free to take on any value, since heights above a certain bound would have affected the measured part of the image had they been present. If the part of the tip which extends beyond the edge is everywhere above this bound, then we know that there were no tip-surface touches there.

If certainty map values near the edge are of interest, we can calculate them with no change in icmap. We need only change the inputs. Here is the recommended procedure: Given an *N* × *M* measured image and *n* × *m* unreflected tip with its zero at pixel (*x*_c_, *y*_c_):

1. Create a new array of size at least (*N* + *n* − 1) × (*M* + *m* − 1).
2. Imbed the measured image in the interior of the new array leaving margins at least *x*_c_ pixels wide on the left and *n* − *x*_c_ − 1 pixels wide on the right, with bottom and top margins of at least *y*_c_ and *m* − *y*_c_ − 1 respectively.
3. Set the value of the pixels in the margins to a large height. A safe choice is a height greater than the maximum height in the measured image plus the range from maximum to minimum in the tip. (But do not use a height too near the maximum allowed by the data type or you risk overflow.)
4. The new augmented image is now the measured image imbedded in an array with high margins. Compute an augmented reconstructed surface from this image using the ierosion routine as before. The margin in this result contains the aforementioned bound above which the unseen part of the specimen cannot lie without affecting the measured part of the image.
5. Compute the certainty map using icmap with the augmented image, augmented reconstructed surface, and tip as inputs. Strip the margins from this result to obtain the certainty map which corresponds to the original (non-augmented) reconstructed surface.

## 5. Blind Estimation of Tip Shape

### 5.1 Blind Reconstruction Equations

In order to reconstruct the specimen from the image, it is necessary to have a 3-d model for the tip geometry. Since tips may abrade or suffer damage during imaging, it is desirable to frequently re-measure their geometry. Optical or electron microscopic methods do not directly provide 3-d information, require removal and reinsertion of the tip, and suffer from their own probe-specimen “convolution” effects [27, 28], even though the probe is a photon or electron. Tip estimation by imaging a known characterizer, as described in Sec. 4.1, does not eliminate the need for an independent determination of a geometry. It simply transfers that requirement to the characterizer.

An alternative is one of the blind tip estimation [18–20] methods. As the name implies, this is estimation using the image of an *unknown* tip characterizer. This author has already published a detailed derivation of one procedure [18] capable of reconstructing tips with complex geometries. The algorithm will be provided and discussed here, but the derivation will not be repeated. Williams et al. arrived at the same result [19]. Dongmo et al. discuss a related method for blind estimation of tips that can be characterized with a small number of parameters [20].

There is a simple explanation of blind reconstruction which serves to provide an intuitive rationale. Practitioners of SPM are well aware that image protrusions are broadened replicas of those on the specimen. However, it is only convention which determines which of the two objects being scanned across one another is the tip and which the specimen. We are equally entitled to regard features on the image as broadened replicas (albeit inverted) of the tip. In particular, for example, it is not possible for the radius of the tip at its apex to be larger than the radius at the top of the sharpest isolated maximum in the image, since this would imply that the corresponding specimen feature had a *negative* lateral dimension. A similar consideration applies to parts of the tip away from the apex and corresponding parts of the image to which they give rise. Since tips are chosen to be slender and sharp, it can safely be assumed that they do not interact with surface objects that are sufficiently far away. In this way, sufficiently separated subsets of the image may be regarded as independent images, each of which places an outer bound on the tip shape. The true tip shape must be inside of the envelope which is at each point equal to the tightest of all these bounds. Reconciling all of the bounds produces the bluntest tip, *P*_R_, consistent with the observed image. Putting it another way, for tips blunter than *P*_R_ there is no *conceivable* specimen which would give rise to the observed image—their surfaces invariably would be required to have some feature with negative width, which is unphysical.

We will need some of the detailed results from Ref. [18] in order to explain the algorithm. There, we described an iteration process:

$$P_{i+1}=\underset{x\in I}{\cap }\left[\left(I-x\right)⊕P_{i}^{\prime }\left(x\right)\right]\cap P_{i}.$$ (14)

Equation (14) allows calculation of the ***i*** + 1st iteration result given the *i*th. The object, $P_{i}^{\prime }$, was defined in Ref. [18] to be *P_i_*′(***x***) = {***d***|***d*** ∈ *P_i_* and 0 ∈ *I* − ***x*** + ***d***}, a definition which we here give in the simpler form,

$$P_{i}^{\prime }\left(x\right)=P_{i}\cap^{\text{​}}\left(x-I\right).$$ (Definition 4)

When this process is continued until convergence, we call the result, *P*_R_.

$$P_{R}=\underset{\left(i\to \infty \right)}{\lim}P_{i}.$$ (15)

We proved that each iteration of Eq. (14) produces a result smaller than or equal to the preceding one, but that each *P_i_* remains larger than the actual tip. This convergence limit is the best estimate of the tip, as obtained by blind reconstruction.

**Fig. 6:**
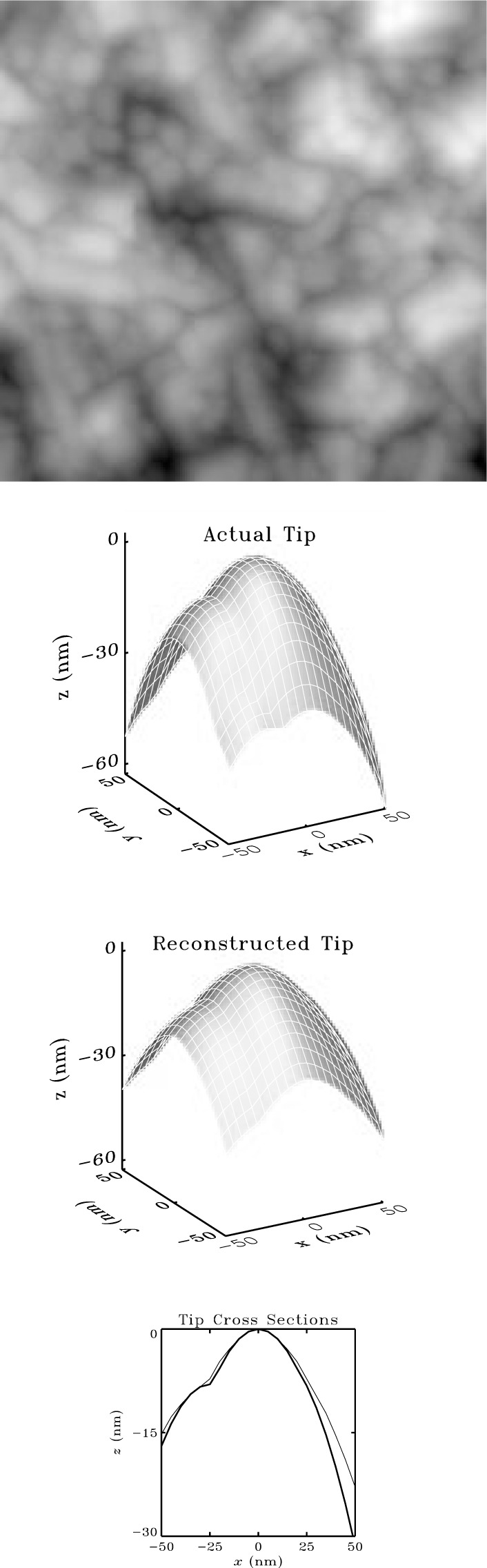
Illustration of results of blind tip reconstruction. A 2 µm × 2 µm simulated surface (a 1 µm × 1 µm piece of which is shown at top), similar to an experimentally observed granular surface [29], was constructed with minimum feature radius 25 nm. An image was computed by dilation with the actual tip (shown), constructed with 40 nm radius at the apex. The blind reconstruction result was then computed by iterating Eq. (14) to convergence, and is shown for comparison with the actual tip. Cross sections through the apex of the actual tip (thick line) and the reconstruction result (thinner line) are compared at the bottom.

Figure 6 illustrates results obtained by blind reconstruction in a simulation. Computer models of a specimen and tip (shown) were constructed, and the image computed from them by dilation. The blind reconstruction result was computed from Eqs. 14 and 15 using the image and a starting outer bound on the tip (a square pillar, flat on the top and ~ 100 nm on a side—see the discussion below) as inputs. (Dimensions in nanometers are supplied in the figure for greater concreteness. The scale is set by the actual granular surface [29] upon which the simulation was based.) The fidelity of the result is typical of cases in which the specimen contains features somewhat sharper than the tip. When the tip is sharper than *all* features on the specimen, the approximation is not as good [18, 21].

## 5.2 Choosing an Initial Upper Bound

All that is required to start the iteration in Eq. (14) is *P*_0_, the initial outer bound. In practice, one typically uses

$$P_{0}=\{\begin{array}{cc} 0 & \text{for}|x|<s_{x}/2\;\text{and}|y|<s_{y}/2\\ -\infty & \text{otherwise} \end{array},$$ (16)

which places the origin at the center of a tip with rectangular cross section of size *s_x_* × *s_y_*. This is the bluntest possible tip of this lateral dimension. The maximum height is 0 in order to satisfy the convention that the apex be at the origin. The dimensions *s_x_* and *s_y_* define the rectangular (chosen for convenience in working with rectangular arrays) “footprint” of the tip. They define a distance outside of which image features may be regarded as arising independently of each other. They should be chosen large enough that points on *P* with lateral coordinates outside of this rectangle do not make contact with the specimen. The choice is often made based on a “back of the envelope” estimate as follows: Suppose our specimen’s topography has 100 nm of relief. Further suppose our tip is nominally parabolic with *z* = *x*^2^/(2*r*) and *r* = 40 nm. Then even for the most unfavorable specimen geometry (i.e., a vertical wall 100 nm high) points on the tip with lateral coordinate (*x*) greater than 90 nm will have *z* > 100 nm and will never contact the specimen. In this case, *s_x_*/2 = 90 should be good enough. To allow for the possibility that the tip is more blunt than the nominal value one typically builds in a margin of safety by increasing the result of such a calculation by some suitable amount.

**Fig. 7:**
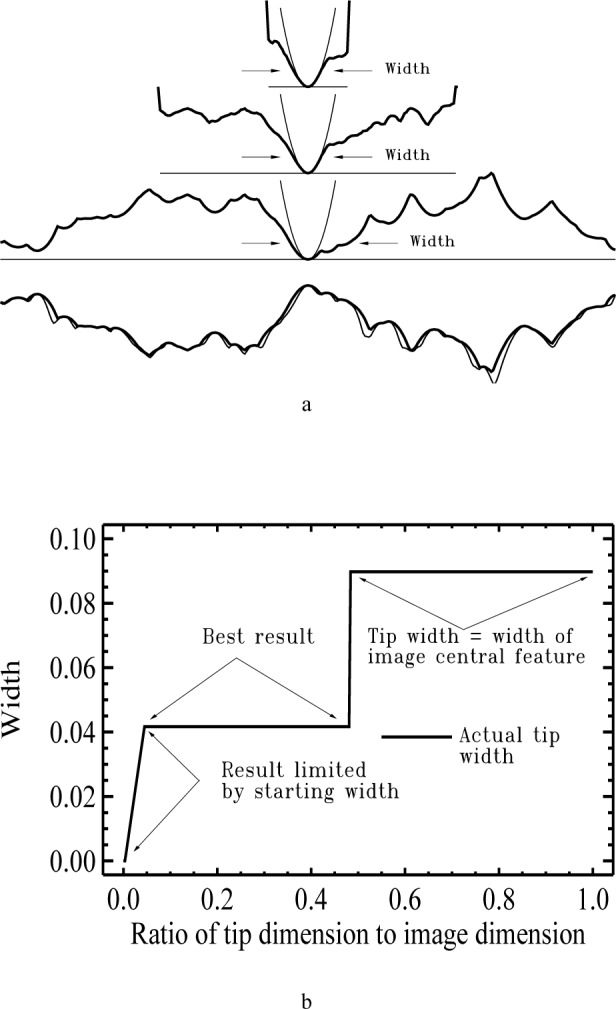
Effect of *s*_x_ [Eq. (16)] on reconstructed tip size. The bottom curves in (a) are an image profile (thick line) simulated by dilation of a surface profile (thinner line) with a parabolic tip. Above are the tips (offset for clarity) produced by blind reconstruction for three choices of starting width, *s_x_*, shown by the thin horizontal lines. The actual tip is also shown for comparison. The height above the apex labelled “width” and indicated by arrows (the same height in each case) indicates the level from which tip widths were computed for comparison in (b). In (b) the horizontal axis indicates *s_x_* as a fraction of the image profile length. The vertical axis indicates the width in the same units.

Figure 7 shows a simulation illustrating some of the considerations for the choice of the lateral dimension. For this example, we use a profile rather than a full 3-d image, so we need only think about the choice of *s_x_*. Because designation of tip and sample is arbitrary, it is always at least a theoretical possibility that the specimen is a sharp spike and the measured image is actually an image of the tip. This possibility is reflected in the lowest of the three reconstructed tips in Fig. 7a, where the tip dimension, *s_x_*, was chosen equal to the dimension of the measured image. In this case, the blind construction method returns *P*_R_ = *I*, as it must. The estimated actual tip is therefore −*I* (the reflection of *I* in both *x* and *z*), as shown in the figure. Such a large starting estimate places no meaningful constraints on the result.

If, on the other hand, we can place a rough limit on the tip shape, we can do much better. We might, for example, know from an optical inspection that the tip diameter is smaller than an optical wavelength, or we might know from electron microscope inspection of tips that the manufacturing process typically produces radii below 100 nm. This would allow us to start with a smaller *s_x_*, using a rough calculation like that suggested earlier. The results from two such smaller starting estimates are shown as the remaining two tips in Fig. 7a. The middle of the three tip results used *s_x_* nearly half the size of the image. The top result used *s_x_* only 13 % of the image size. Nevertheless, the two results are nearly identical near the apex.

This lack of sensitivity to the choice of *s_x_* is illustrated in Fig. 7b which shows a plot of the reconstructed tip width near the apex as a function of *s_x_*. For large *s_x_*, corresponding to the first tip in Fig. 7a, the width is that of the tallest peak in the image. This peak is asymmetric, with smaller secondary peaks on one side. These secondary peaks might be due to actual secondary features on the specimen, or they might be features of the tip. At this stage it is not possible to eliminate the latter possibility with the result that *P*_R_ is broad. As *s_x_* is reduced, a point is reached at which the other peaks in the image are considered to provide independent information about the tip. Some of these do not contain the same secondary structures as the first peak, thereby eliminating the possibility that they are associated with the tip. At this point the width decreases suddenly to a value close to the correct one. This result is maintained for a large range of *s_x_* values. The two upper, more symmetrical, reconstruction results in Fig. 7a come from this region. Only when *s_x_* becomes smaller than the width of the actual tip do we reach a region where the result is limited by *s_x_*.

Some general features of blind reconstruction are illustrated by this. Except for the case when the starting footprint is too small to permit representation of the actual tip, all of the results, whatever the choice of *s_x_*, were valid outer bounds on *P*. As long as the footprint is larger than the actual one, smaller is better since it allows division of the image into a larger number of independent pieces, each of which supplies information about *P*. The result tends to change discontinuously as the footprint is reduced. This is because not all tip shapes are consistent with a given image. When a starting out bound is provided, the resulting reconstruction “snaps” to the next smallest size which is consistent. This is important to the utility of the method. If the result changed smoothly with changing *P*_0_, we would never be sure whether we had gotten the answer right. As it is, the result provides significant improvement to the starting estimate and is insensitive to the chosen *P*_0_ within broad ranges.

## 5.3 Blind Reconstruction Algorithms

Appendix E contains algorithms needed to estimate a tip from an image by blind reconstruction. There are three primary routines. The first computes the largest tip consistent with a single given point on an image. The second iterates the first through all image points until convergence. The third iterates through only a subset of specially chosen points in the image. These routines all include a parameter called “thresh” among their inputs. We postpone discussion of this parameter until our discussion of noise in Sec. 6, only remarking for the time being that thresh = 0 corresponds to the equations given so far.

### 5.3.1 Tip Estimation From a Single Image Point

Sec. 13.1 contains a listing for itip_estimate_point(). This function calculates [(*I* − ***x***′) ⊕ *P_i_*′ (***x***′)] ∩ *P_i_* for a single point, ***x***′. This is the right-hand side of Eq. (14) and the basic building-block for all of the tip estimation routines which follow.

In the middle of the routine are two blocks of code, one from line 172 to 180, the other from line 190 to 204. These calculate *T* [(*I* − ***x***′) ⊕ *P_i_*′ (***x***′)] (*x*, *y*) for a given image pixel at ***x***′. The first block does this if ***x***′ is in the interior of the image, where edges are not an issue. The second block is used when ***x***′ is near the edge. Once this term is determined, its intersection with *P_i_* is formed (at line 182 or 206, depending on the block) completing the calculation of the right-hand side of Eq. (14) for that particular tip pixel. The outer loops complete the calculation for all tip pixels, ***x***.

To understand the details of the inner blocks, it is simplest to leave edge issues aside at first and consider lines 190 to 204. We use Definition 2 (for dilation) and Properties 1 and 2 to write

$$\begin{array}{l} T\left[\left(I-x^{\prime }\right)⊕{P_{i}}^{\prime }\left(x^{\prime }\right)\right]\left(x,y\right)\\ =\underset{\left(d_{x},d_{y}\right)}{\max}[i\left(x+x^{\prime }-d_{x},y+y^{\prime }-d_{y}\right)\\ +p\left(d_{x},d_{y}\right)-i\left(x^{\prime },y^{\prime }\right)] \end{array}$$ (17)

We now discuss the meaning of this equation in terms of a practical implementation, where the image is represented by an im_xsiz × im_ysiz array and the tip by a tip_xsiz × tip_ysiz array. The coordinates *d_x_* and *d_y_* range over the domain of *P*′. That is, they are essentially *tip* coordinates, addressing the intervals [0,tip_xsiz) and [0,tip_ysiz), except that Definition 4 places an additional condition, about which more shortly. The coordinates *x*′ and *y*′ are *image* coordinates, ranging over the intervals [0,im_xsiz) and [0,im_ysiz). Finally, we anticipate that our next step [see Eq. (14)] will be to form the intersection of Eq. (17)’s result with the current best estimate, *P_i_*, of the tip. Therefore, only values of *x* and *y* in the range [0,tip_xsiz) and [0,tip_ysiz) need be calculated.

Do we need to make some accommodation, as we did for the dilation and erosion algorithms, for the fact that Eq. (17) was derived for tips with apex at the origin while our C arrays are addressed with (0, 0) at the corner? The answer is, in principle yes. However, perhaps surprisingly, it makes no difference this time. Both the left-hand side of Eq. (17) and p() on the right are tip arrays, the arguments of which must range over all or part of [0,tip_xsiz) and [0,tip_ysiz), as already mentioned. The correction for placing the tip apex at (*x*_c_, *y*_c_) instead of (0, 0) would be to replace (*x*, *y*) and (*d_x_*, *d_y_*) with (*x* − *x*_c_, *y* − *y*_c_) and (*d_x_* − *x*_c_, *d_y_* − *y*_c_) in the remaining terms. However, (*x*, *y*) and (*d_x_*, *d_y_*) either do not appear in the remaining terms or appear in pairs with opposite sign, cancelling any offset. As a result, line 177, which calculates the term in Eq. (17)’s square brackets, contains no explicit offsets.

We have so far glossed over the conditions placed on ***d*** by Definition 4. We consider them now. Definition 4 requires the apex, then contemplated as being at the origin, to be contained within *I* − ***x*** + ***d***. Since it is convenient for programming to place the apex at ***d***_c_ = (*x*_c_, *y*_c_, 0) the same condition on the apex becomes ***d***_c_ ∈ *I* − ***x***′ + ***d***. Switching from set notation to surface functions, this becomes

$$\begin{array}{l} 0\leq i\left(x^{\prime }-d_{x}+x_{c},y^{\prime }-d_{y}+y_{c}\right)\\ -i\left(x^{\prime },y^{\prime }\right)+p\left(d_{x},d_{y}\right)-p\left(x_{c},y_{c}\right), \end{array}$$ (18)

or since we retain the condition that the tip’s apex, *p*(*x*_c_, *y*_c_), be zero height

$$\begin{array}{l} i\left(x^{\prime },y^{\prime }\right)-i\left(x^{\prime }-d_{x}+x_{c},y^{\prime }-d_{y}+y_{c}\right)\\ \leq p\left(d_{x},d_{y}\right). \end{array}$$ (19)

In the code, the first part of the condition in Definition 4 is enforced by restricting the (*d_x_*, *d_y_*) loop beginning at lines 174 and 175 to the interval [0,tip_xsiz), [0,tip_ysiz). The second part is enforced at line 176, which evaluates Eq. (19) and skips to the next (*d_x_*, *d_y_*) if it is not true. The (*d_x_*, *d_y_*) loop computes the maximum only of those terms meeting these conditions, thus completing the evaluation of Eq. (17) when (*x*′, *y*′) is not too near the edge of the image.

When it is near the edge, as always, additional care is needed. The general philosophy in dealing with unknown parts of the image is to assume the worst case. In calculating *P*_R_ we are computing an upper bound on *P*. Therefore, we *never* revise a tip pixel’s height downward if there exists *any* conceivable configuration of the image in the unknown area beyond the edge which would be consistent with the pixel’s present value.

**Fig. 8:**
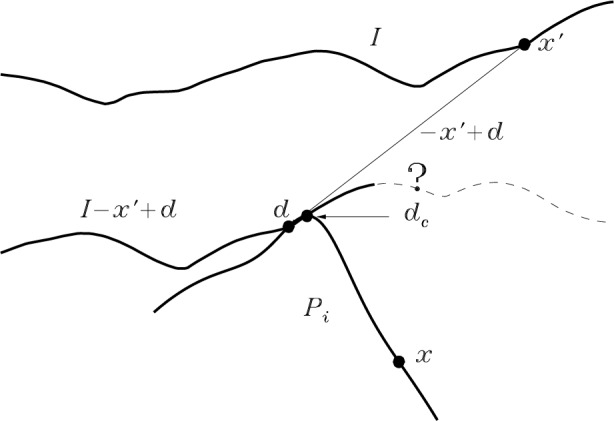
When ***x***′ is near the edge of the image, *I*, part of *P_i_*, which may include the apex at ***d***_c_ and/or other points like the one at ***x***, may lie over the edge once the image is translated (*I* − ***x***′ + ***d***). The unknown part of the image is suggested by a dashed line with question mark.

Algorithmically, the problem of edge proximity chiefly manifests itself via the fact that the indices into image [] [] might take on values outside the allocated memory space for that array either in line 176 or 177. Physically, this corresponds to the situation illustrated in Fig. 8. When ***x***′ is near the edge of the image, there may exist some values of ***d*** such that when *I* is translated by ***d*** −***x***′ the point ***x***, which we require for forming the intersection with *P_i_*, or the image apex at ***d***_c_, which we require for evaluating the condition in Eq. (19), or both, lie outside of the known part of the image. The code block between lines 190 and 204 is essentially a repetition of the one we just considered, but with additional lines interspersed to handle the various cases which may arise.

To begin with, we can subdivide all the possibilities into six (2 × 3) relevant cases. These correspond to two possibilities for the point ***x*** and three for the apex location. The lateral coordinates of ***x*** either do or do not lie within the domain of the translated image. We call these possibilities “***x*** inside” and “***x*** outside.” If the lateral coordinates of ***d***_c_ lie inside the domain of the image and the vertical coordinate lies on or below the translated image surface [condition given by Eq. (19) is true], we say that ***d***_c_ is “inside.” If the vertical coordinate is above the translated image surface [Eq. (19) is false] ***d***_c_ is “outside.” If the lateral coordinates of ***d***_c_ are outside the domain of the image [impossible to evaluate Eq. (19)], then the status of ***d***_c_ is “indeterminate.”

We can simplify these six cases to four by realizing that it is appropriate to treat ***d***_c_ indeterminate as equivalent to ***d***_c_ inside. That is, when ***d***_c_ falls outside the known area of the image, the worst case is to assume that the image height is sufficiently large that Eq. (19) is satisfied. This can only result in the dilation having a larger value, with corresponding smaller reduction in the current tip estimate when the intersection is formed.

The appropriate action to take depending upon the four remaining possibilities follows. Possibilities 1 and 2: When ***d***_c_ is outside and ***x*** either inside or out, then ***d*** ∉ *P*′ (***x***′). We therefore ignore this configuration and go to the next value of ***d***. Possibility 3: When ***d***_c_ is inside and ***x*** is outside, we must assume, worst case, that *i* + ***x*** − ***d*** → ∞. Since the (id, jd) loop is computing the maximum value of this quantity, there is no need to continue the loop—we will not subsequently find a value larger than infinity! We therefore abort the loop, making no change in the tip estimate for this ***x***. Possibility 4: When ***d***_c_ is inside and ***x*** is inside, we have the “normal” case that we already treated in the interior.

### 5.3.2 Full Tip Estimation Algorithm

To extract all of the available information about the tip shape, we would like to apply itip_estimate_point() to all points in the measured image. The routine, itip_estimate_iter(), in Sec. 13.2 essentially does this. Some of the points can be skipped, however, because we can predict in advance that they result in no refinement of the tip shape. These points are those at which *I* = (*I* ⊖ *P_i_*) ⊕ *P_i_* (see Ref. 18). The time saved by avoiding calls to itip_estimate_point() for those points at which this is true usually provide a generous return for the time invested calculating (*I* ⊖ *P_i_*) ⊕ *P_i_*.

The routine, itip_estimate(), also in Sec. 13.2, repeatedly calls itip_estimate_iter() until convergence. This result is *P*_R_ [Eq. (15)]. The input parameters for itip_estimate() are the measured image and its dimensions, the dimensions of the tip to be calculated, the coordinates within this array at which the apex is to be placed (usually the center, but offsetting the apex to one side may be desirable, for example, if one anticipates an asymmetrical tip), and a pointer, tip0, to a starting tip estimate. The starting estimate is often simply an array of the appropriate size filled with zeros, but it may be the result of a previous partial calculation. (See the next section.) The result of itip_estimate() replaces the original values in tip0.

### 5.3.3 Partial Tip Estimation Algorithm

Section 13.3 contains a partial tip estimation algorithm, itip_estimate0(). This one forms the intersection of itip_estimate_point() applied only to a subset of image points. While not as complete as the full algorithm, it can be calculated in substantially less time. By choosing those image points which are likely to contain the most information about the tip, the result of this partial calculation is often quite good. It may be used as the final tip estimate, or it may become, as its name suggests, a starting estimate for the full tip estimation routine, thereby reducing the total time required for the full calculation.

The algorithm employed here selects points which are local maxima in the image. Alternatives are possible, for example choosing points on the image with high curvature. The routine, useit(), sets the criterion for points used by itip_estimate0(). Programmers can change the criterion simply by changing this algorithm.

## 6. Noise and Other Limitations

We have heretofore ignored the effect of noise. Many measuring instruments in common experience are at least approximately linear. As soon as one begins to ask questions about probe/sample interactions in the SPM, however, one is dealing with an inherently nonlinear interaction. This results in a different, perhaps less familiar and therefore less intuitive, effect of noise upon such operations as surface reconstruction and tip estimation.

### 6.1 Effect of Noise on Surface Reconstruction

Any measuring instrument can be conceptualized as producing a measured output, $\hat{o}$, from the input, ***x***, via some instrument dependent measuring operator, *M*, so that ideally

$$\hat{o}=M\left\{x\right\}.$$ (20)

In the familiar linear case, one can write this as a convolution of the input with an “instrument function” or in Laplace transform space as a product of the input with an instrument “transfer function.” *M* has an inverse, $x=M^{–1}\left\{ô\right\}$ which allows “reconstruction” of the input. If there is noise on the output ( ${ô}_{m}=ô+n$ where *n* is a noise term characterized, perhaps, by average value 0 and standard deviation *σ*) then

$$M^{-1}\left\{{\hat{o}}_{m}\right\}=M^{-1}\left\{\hat{o}+n\right\}=x+M^{-1}\left\{n\right\}.$$ (21)

Thus, noise on the output can be referred back to the input as an equivalent input noise, *M*^−1^{*n*}. Furthermore, *M*^−1^ is linear, so if the average of *n* is 0, so is the average of *M*^−1^{*n*}. This means that noise does not *bias* the reconstruction. One may either average the results of many reconstructions or reconstruct the average of many measurements. The results are the same.

**Fig. 9:**
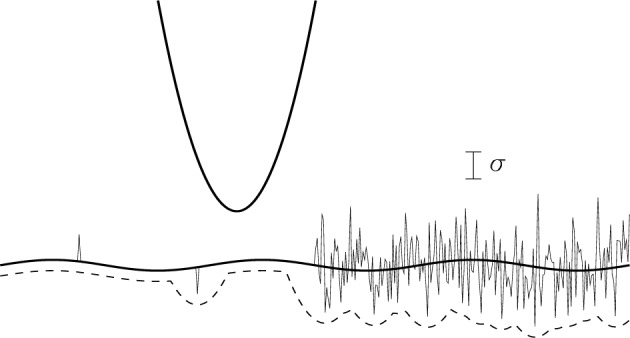
Effect of noise (one standard deviation, *σ*, indicated) on surface reconstruction. Shown are a parabolic tip and a noisy image (thin line) superimposed on the actual surface (thick line). The reconstructed surface (dashed line) is offset slightly for clarity.

This familiar, almost intuitive, behavior applies only to *linear* instruments. In particular, it does *not* apply to surface reconstruction in SPM. This is illustrated in Fig. 9. The thick wavy solid line is a surface on which has been superimposed a noisy image (the thinner line). For illustrative purposes, the left half of the image has only two noise spikes, an upward-going one and a downward-going one. On the right, all pixels are noisy, with one standard deviation (henceforth designated *σ*) indicated. The dashed line is the erosion of the tip from the noisy image, offset slightly for clarity. It is evident that the upward-going spike on the left had virtually no effect on surface recovery. Remember that erosion is taking a minimum envelope (see Fig. 4). The upward spike has little effect because the adjacent pixels, coupled with the broad tip, are enough to establish that the specimen could not have been that high. The effect of the downward-going noise spike, however, is magnified. It manifests itself as a tip-shaped depression in the result.

On the right of the image, where the noise has a wavelength short compared to the tip, the likelihood of encountering a negative noise spike within an area comparable to the tip size approaches one. The reconstruction height is therefore almost always smaller than the actual specimen height. The amount by which it is smaller depends upon the size of the tip and the frequency characteristics of the noise. For example if the noise is Gaussian, and if the noise level at each pixel is independent of its neighbors, then we should expect to find that ~ 1/3 of pixels deviate from the mean by more than 1*σ*, 5 % by more than 2*σ*, 0.3 % by more than 3*s*, and so on in the familiar Gaussian progression. If the tip effectively interacts with the specimen over a 10 × 10 pixel square area, we should not be surprised to see events occurring within these 100 pixels that have an individual probability of only 1/100. Thus a bias of 2*σ* or even 3*σ* would be expected. In Fig. 9, the bias is nearly 2*σ*. Fortunately, Gaussian probability distributions have exponential tails, so multiples of *σ* much greater than 3 or 4 should be uncommon.

As a consequence of this bias, smoothing or filtering the reconstruction result is not equivalent to smoothing the image and then reconstructing. The latter is generally to be preferred. Even so, filtering cannot be expected to remove *all* of the noise. It is therefore necessary to be aware that noise introduces bias to the extent of some small multiple of the remaining rms noise level.

### 6.2 Effect of Noise on Certainty Maps

**Fig. 10:**
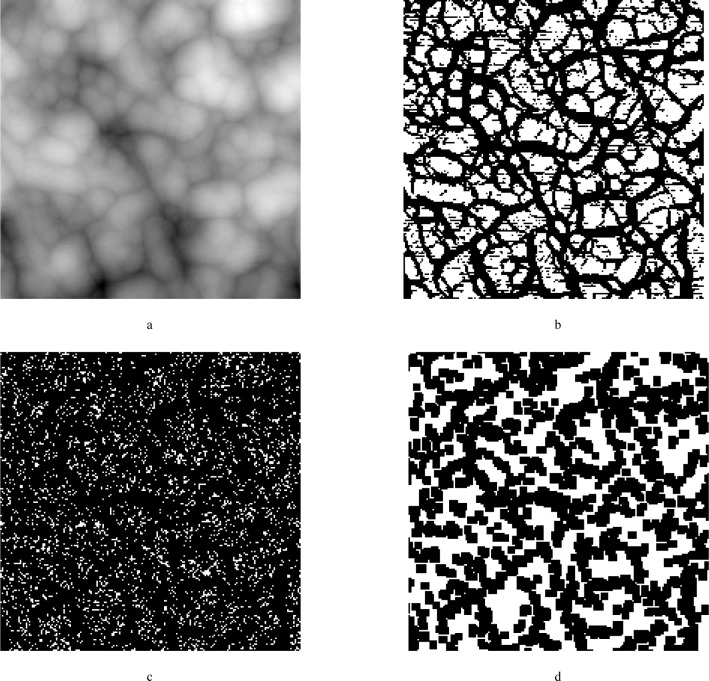
Effect of noise on certainty map. (a) An image simulated with a parabolic tip. (b) The certainty map upon reconstruction of the noiseless image. White areas are those scored as recoverable. (c) The certainty map upon reconstruction of image + noise. (d) Closing small gaps between pixels in (c) as an aid to visualizing areas with a higher density of points.

Noise has a more profound effect on the Pingali certainty maps described in Sec. 4.3. Figure 10a shows part of a simulated image with a vertical scale spanning approximately 160 nm. A random number generator has been used to add Gaussian noise with *σ* = 1 nm. Figure 10b shows the correct or ideal certainty map obtained during reconstruction of the noiseless image. By contrast, Fig. 10c shows the results when the noise is included. Though Figs. 10b and c resemble each other the correlation coefficient is only 0.2.

The source of the problem is evident in Fig. 9. The reconstruction of noisy images contains many tip-shaped depressions resulting from the deeper negative noise spikes. These tip-shaped regions will all be scored as nonrecoverable by a test that counts the number of pixels touched by the tip. Thus, even in places where recovery is reasonably good, few pixels will meet this rigorous test.

In the noisy recovery the areas scored as recoverable are far less dense than in the noiseless recovery. This suggests that we could improve the result by scoring areas of Fig. 10c according to whether or not they are in a high density neighborhood. We could do this either with a density plot or by closing gaps between pixels when the gap size falls below some threshold. The noiseless certainty map had the appealing property that there were no false positives. A closing or density plot will no longer have that property, but for noisy reconstructions may give an improved qualitative measure of the confidence to be placed in the result. Figure 10d creates such a “confidence” map from the result in (c) using the closing method. The correlation coefficient between the ideal result in Fig. 10b and the result with noise in Fig. 10d is 0.4. Unfortunately, performance degrades rapidly with increasing noise, so certainty or confidence maps require more work if they are to be useful at noise levels much greater than that shown here.

### 6.3 Effect of Noise on Blind Tip Estimation

Blind tip estimation, as presented so far, is based upon the assumption that all image features derive from the dilation of the specimen surface with a tip. To the extent that this is true, sharp parts of the image require a correspondingly sharp tip. It was this observation, carried to its logical conclusion, that enabled us to estimate the tip shape from the image.

In fact, however, the assumption is only approximately true. Electronic or vibrational noise often manifests itself as sharp spikes, sharper than the tip which produced the image. The typical result is that in the early stages of the iterative process that ultimately determines *P*_R_ the conclusion is erroneously reached that the tip apex contains a feature of height and sharpness similar to some of these noise spikes. If that were the extent of the effect it would not particularly pose a problem. It is not unusual, it is in fact to be expected, that noisy inputs lead to noisy outputs. However, the error made in the early stages of the iterative process propagates to later stages and is magnified. The too-sharp tip no longer appears consistent with *other* features on the specimen, including some which actually were produced by dilation with the real tip. The algorithm as presented so far responds to even small inconsistencies of this sort by narrowing the tip still further. The overly sharp apex feature in this way propagates away from the apex through subsequent iterations, with the result that the error in the final result can be substantially larger than the noise level.

**Fig. 11:**
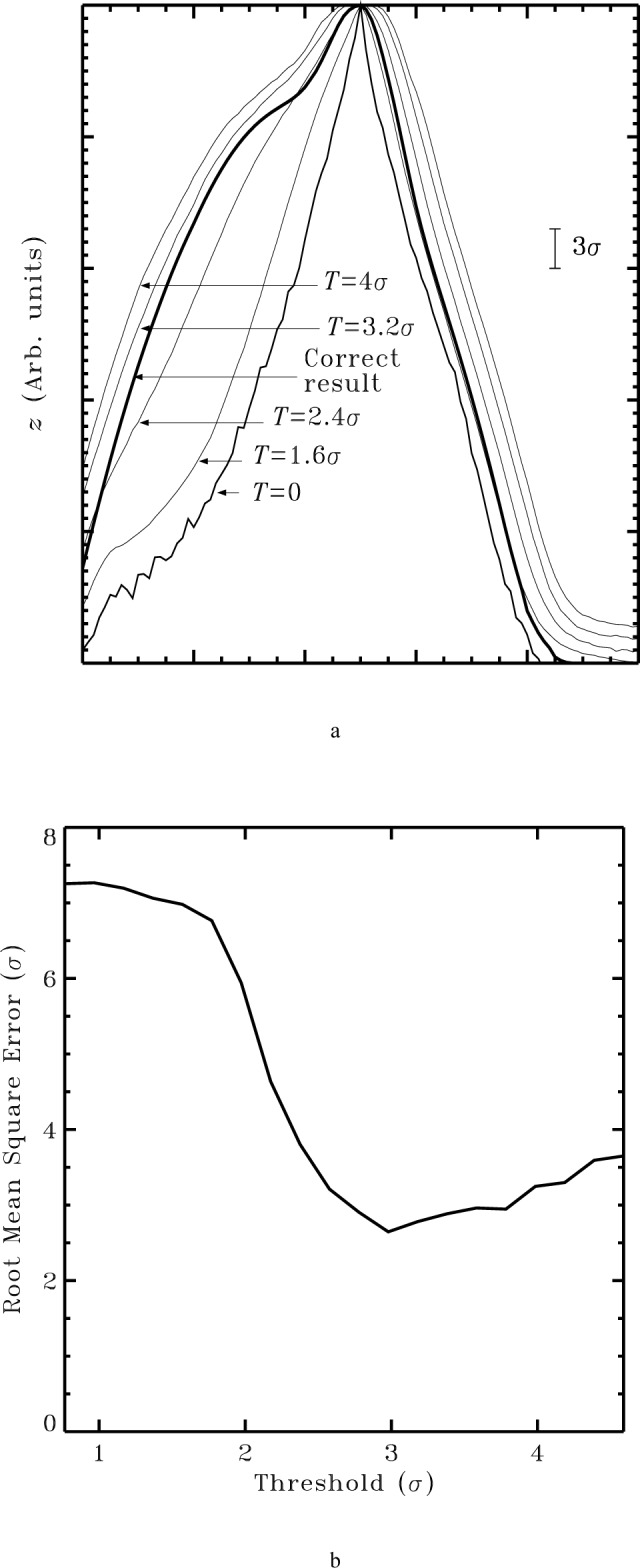
Effect of noise on blind reconstruction as a function of the threshold parameter, *T*. (a) A family of tip shapes constructed from a simulated noisy image (rms noise = *σ*, 3*σ* level as indicated), compared to the ideal result (thickest line) calculated by blind reconstruction of the image without noise. (b) The rms deviation of the computed tip shapes from the ideal result as a function of threshold. Both axes are expressed in units of *σ*.

This problem is illustrated in Fig. 11a. The thickest line, labelled “Correct result,” was obtained by blind reconstruction of a noiseless image simulated by the dilation of a surface with a tip. The other results were all obtained after adding noise to the image (3*σ* level shown). The innermost tip, labelled “*T* = 0,” is the result of a blind reconstruction using itip_estimate() with the threshold parameter set equal to 0. It is considerably sharper than the ideal result. It is very close to *I* ⊖ *I*, which has been shown to be the largest tip which produces no distortion of the surface at all [18]. The repair for this problem which has been implemented in itip_estimate_point() is to introduce a threshold parameter. This parameter, in effect, establishes a level of inconsistency between the image and the tip estimate which will be tolerated. The threshold is implemented in itip_estimate_point() at lines 182 and 206. These are the lines at which the intersection between the current tip estimate and the result of the preceding calculation is formed. When thresh = 0 these lines simply replace the value of the current estimate with the new result if the new result is smaller. When thresh ≠ 0 there is a bias in favor of retaining the current estimate. Only if the difference between the new value and the old one exceeds the threshold is any change at all made, and then not by the full amount of the difference. By replacing the old value with the new one + thresh, we introduce a positive bias intended to offset the tendency, which we saw in Fig. 9, for noise to bias the results negatively.

Results for various settings of the threshold parameter are shown by the remaining curves in Fig. 11a. The rms difference between these curves and the correct (noiseless) result are shown in Fig. 11b as a function of the threshold value. Although this figure is the result for a particular choice of image, tip, and noise, its features are typical. The curve has a minimum, in this case at a threshold near 3*σ*. The location can be understood in general terms. The reconstructed tip shape is determined by some number, *n*, of image pixels with independent noise levels. By inverting the normal probability distribution (the same argument we employed to understand the amount of bias in the erosion of noisy images near the end of Sec. 6.1), if 100 < *n* < 10^5^ we should expect to find some pixels with sampling errors in the range 2.3*σ* to 4.3*σ*. It is therefore to be expected that the best choice of threshold also falls in this range, though it may be higher if other error sources are more important than noise (see Sec. 6.4).

When the threshold is optimum, the difference between the result with noise and the noiseless one is characterized by rms value comparable to the threshold. That is, this rms difference is also typically in the range of 2*σ* to 4*σ*. As we saw in Sec. 6.1 this is the same sort of error one encounters with simple erosion of noisy objects. Thus, with the use of the threshold parameter, the effect of noise in blind reconstruction is similar in magnitude to its effect in tip reconstruction by simple erosion with a known characterizer.

The deviation of the tip from the ideal result increases to either side of this minimum, to the left because the result is too sharp and to the right because it is too blunt. The increase to the left is much more rapid than that to the right. This also is typical. As the threshold is increased from 0, the transition from too sharp to optimum happens relatively suddenly. Continued increase of the threshold value past its optimum point then results in a gradual deterioration of the quality of the result.

### 6.4 Other Limitations

Electronic and vibrational noise are not the only phenomena which can introduce into an image features that are not the result of dilation of the specimen with a single tip geometry. Others include scanner nonlinearities, flexing of the cantilever or tip as a result of friction or other lateral forces [30], feedback loop overshoot resulting from scanning too quickly, mid-image tip changes due to collision with the surface, and, at the sub-nanometer level, failure of the standard imaging model due to inhomogeneous sample compressibility or work function.

These are *possible* sources of trouble. The extent to which they will be important in practice is still largely unexplored. With a threshold of 0, any of these phenomena, even at low levels, might be expected to cause the same sort of instability in the tip reconstruction algorithm produced by noise. However, if the threshold is sized comparably, the algorithm will stably produce a result. Of course, the accuracy of that result degrades with increasing threshold, so the important thing will be the size of these effects relative to the desired accuracy of the reconstruction. Should they prove to be a problem, there are methods, still largely unused, to improve the performance of the instruments. Scanner nonlinearities may be overcome through the use of closed-loop operation around linear position sensors. These methods are beginning to be used in instruments designed for length metrology [5, 6, 31]. If lateral forces are strong enough to cause cantilever flexing, there are imaging modes which minimize friction [32] and even AFM’s which operate without cantilevers [33]. Feedback loop overshoot can be combatted by slowing the scan speed, at least near steep specimen features. Tip changes can be detected by doing tip characterization both before and after imaging important specimens. Efforts are now underway to verify the operation of blind reconstruction experimentally.

## 7. A Practical Guide

This section is intended to be a user’s guide to the software provided in the appendix. It includes typical examples of usage, guidelines based upon experience, and indications of common problems.

### 7.1 Filtering

Dilation, erosion, and blind reconstruction of tips are all nonlinear operations. As we noted at the end of Sec. 6.1 they do not commute with filtering operations. For example, filtering an image followed by erosion in general produces a different result than erosion followed by filtering. Since morphological operations tend to exaggerate certain types of noise, it is advantageous to first filter the data.

Because images are raster scanned, low frequency noise manifests itself as long wavelength distortions in the raster direction but short apparent wavelength in the orthogonal direction, resulting in the familiar streakiness of many SPM images. This is usually removed in an image flattening step which includes, at least in part, a line-wise component. A method for combining an area-wise surface fit with line-wise flattening in a least squares approach has recently been proposed [34]. Inclusion of linewise flattening is particularly important when performing a blind tip reconstruction, for otherwise the sharp steps from one line to the next might be mistaken as indicating similar sharp features on the tip. For the same reason care must be exercised in performing the background fits only over those portions of the image that truly represent background. For example, in flattening an image of a biomolecule on a flat background, the molecule should be excluded from the fit. Otherwise the flattening algorithm may itself introduce just those sorts of sharp line to line transitions which we seek to avoid.

After flattening a variety of filtering options are available. Among the most common are neighborhood averaging, with or without weighting, and median filtering [35]. Neighborhood averaging smooths edges in an image, including real ones. For this reason median filters are often preferred [36] despite the fact that they require more computation time. Actual sharp features in the image contain much of the information about the tip shape which the blind estimation procedure extracts. Preservation of the real ones is therefore just as important here as avoidance of artificial ones was in the last paragraph. For that reason neighborhood averaging filters should be avoided.

### 7.2 Image Simulation and Surface or Tip Reconstruction Using Dilation and Erosion

#### 7.2.1 Image Simulation

Occasions for image simulation often arise in a straightforward way. For example, we may have a structural model for a biological molecule based upon theory or previous experiment. We have an image believed to be an image of this molecule, and an estimate of the tip shape when the image was taken. Is the actual image consistent with the expected image? To answer this question, one constructs a computer model of the molecule on a flat substrate (*S*) and another model of the tip (*T*). Invert the tip using ireflect to obtain *P* = − *T*. Then use idilation with *S* and *P* as inputs to obtain *I*.

#### 7.2.2 Surface or Tip Reconstruction

Reconstruction of the surface from a measured image once a tip estimate is available is similarly straightforward. One simply uses the ierosion() routine with *I* and *P* as inputs. Alternatively, as we have mentioned, if we have a known reference surface we can use ierosion() with *I* and *S* to determine *P*_r_. Remember that this *P*_r_ is equal to *P* only for those parts of *P* which touched the characterizer. Elsewhere *P*_r_ is an outer bound. Of course, errors in characterizing the reference surface propagate and produce an error in the tip estimate.

**Fig. 12:**
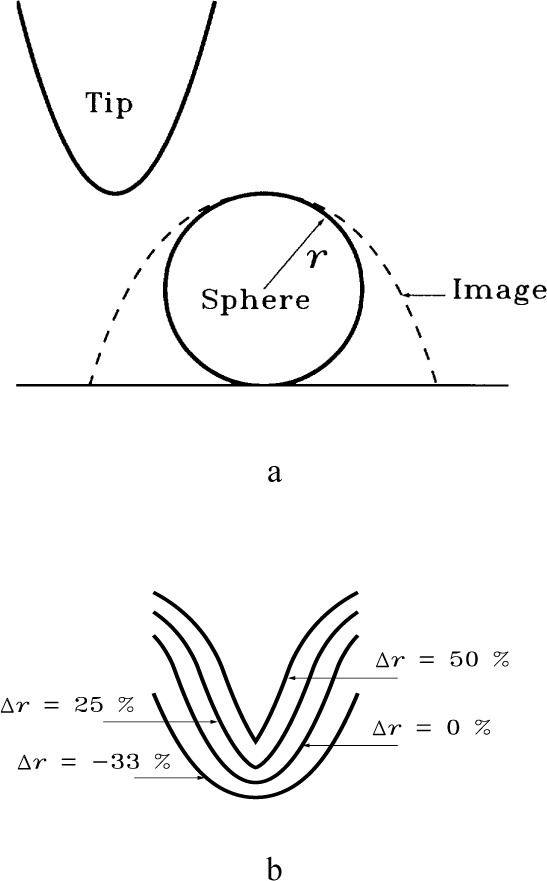
Using a spherical tip characterizer. (a) The image (dashed line) produced when a spherical object is scanned with a tip. (b) Tips, shown offset for clarity, reconstructed by eroding spheres of various sizes from the image in (a). The spheres differ from the actual characterizer radius by amounts varying from − 33 % to + 50 %, as indicated.

One characteristic failure, illustrated in Fig. 12, is easy to recognize. In Fig. 12a we show a spherical tip characterizer and its image. In Fig. 12b are four tips determined by eroding spheres of various radii from the image in Fig. 12a. The Δ*r* = 0 tip nicely reproduces the actual tip. If uncertainty in the characterizer radius leads us to erode too large a sphere (the Δ*r* = 25 % and Δ*r* = 50 % curves) the resulting tip has a characteristic unphysical discontinuity in its slope at the apex and the height at the apex differs from zero. Recognizing this may sometimes give us an indication that something is wrong. Unfortunately, if we err with *r* in the other direction there is no such easy indication. There is nothing unphysical about the Δ*r* = − 33 % result.

### 7.3 Tip Estimation Using Blind Reconstruction

In this section we first make some general comments applicable to blind reconstruction, then consider several modes in which the algorithm may be employed.

The blind tip estimation routines all require the following inputs: an image and its dimensions, the array dimensions of the desired result, the coordinates of the apex within the tip array, a set of starting height values, and a threshold value. The image and its dimensions are usually supplied by a measurement. Reasonable choices for the remaining values must then be supplied.

The array dimensions, tip_xsiz and tip_ysiz, of the desired tip estimate must be chosen large enough to allow description of all parts of the tip that participate in the imaging. In practice, one can often begin by using the lateral extent of some of the taller protrusions in the image as a guide, or by choosing a size calculated based on the expected tip shape, with some extra added for a margin of safety, as described in Sec. 5.2. As we discussed there, the results are not strongly affected if these are chosen a bit too large. However, execution time is adversely affected by the size of the tip estimate. The apex coordinates, xc and yc, are nearly always put in the center of the tip array at tip_xsiz/2 and tip_ysiz/2. It is only rarely justified to populate tip0 with any height values other than zeros at the beginning of a calculation. The tip0 parameter is primarily useful in allowing a partial result, for example from itip_estimate0() or itip_estimate_point(), to be used as a starting point for subsequent refinement.

The correct threshold parameter is more problematical. In principle, one ought to be able to measure the noise level in an image, either by repeatedly imaging the same area or by recording a “noise” image with the lateral scan turned off. A threshold of 3 to 5 times the rms noise, depending upon the size of the image, should be sufficient. In practice however, other factors like imaging errors due to scanner nonlinearities, feedback overshoot, or cantilever bending may be more important than electrical and mechanical noise. The size of these effects may be difficult to estimate a priori. However, it is also observed that the effect of too small an estimate of threshold is to produce a tip that resembles *I* ⊖ *I*, as in the low threshold curves shown in Fig. 11a. This result is unphysically sharp, characterized by a discontinuity in the slope at its apex. For many images, the result transitions sharply to something more reasonable when the threshold reaches the correct value. This can be used to select a threshold by trial and error.

Once starting values for the parameters have been obtained, one could simply insert them into the itip_estimate() procedure to obtain the result. However, one can often save on computation time by using itip_estimate() only as the finishing step of a two step process. The two steps are as follows: (1) Call itip_estimate0(image,…,tip,…). The tip array now holds a partial tip estimate based only on image points pre-selected as most likely to contain significant tip information. Because of this, tip is now often close to the final value. (2) Call itip_estimate(image,…,tip,…). By virtue of lines 232 and 235 this routine will be able to eliminate many steps which would have been required had tip not been pre-refined by step 1.

There are several different measurement modes in which we might employ blind reconstruction. These modes include using the unknown specimen surface as its own characterizer, using a separate characterizer, finding the best tip estimate consistent with several images, and combining blind reconstruction with the erosion method when part of a characterizer is known. Each of these is now considered in turn.

#### 7.3.1 Using an Unknown Surface as its Own Tip Characterizer

With the previously existing methods it was necessary to image a known tip characterizer in addition to the unknown specimen. Since with blind reconstruction a known characterizer is not necessary, the question naturally arises whether we may use the unknown specimen as its own tip characterizer.

**Fig. 13:**
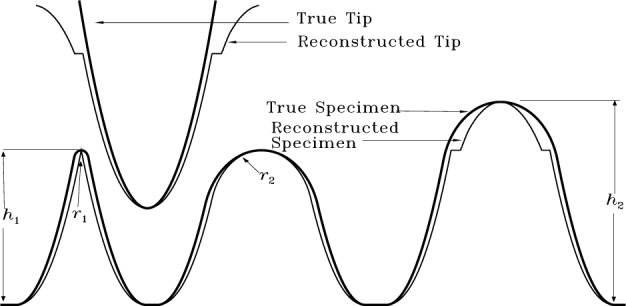
Using a specimen as its own tip characterizer. Parts of the specimen, like the sharp feature at the left or tall one at the right, which participate in defining the tip estimate are not well approximated. Blunter and shorter features like the middle one are.

The answer is a qualified yes, as illustrated in Fig. 13. Here the specimen contains three significant features, a small radius one on the left, a larger radius one of the same height (*h*_1_) in the middle, and a taller (height = *h*_2_) large radius one on the right. Errors in the reconstructed tip for a distance *h*_1_ from the apex are comparable to the small radius feature at the left. As a result, errors in the reconstruction of that feature are comparable to its size—its apex gets eroded to a point. This is not a good approximation. On the other hand, a blunter feature like the middle one is reproduced with errors small compared to its size. This is a reasonable approximation. The taller feature at the right falls in between these two cases. The part of the tip more than *h*_1_ from the apex was untouched by the sharp specimen feature at the left. The only information concerning this part of the tip comes from the tall feature itself, parts of which are therefore not well approximated.

The conclusion from this illustration is that when a specimen is used as its own tip characterizer, some parts of the specimen (those which are sharpest or tallest and therefore participated in defining the tip shape) are not well reconstructed, but other parts are. Therefore, this method can be usefully employed when the features of interest on the specimen are something *other* than the sharpest or tallest ones contained there.

#### 7.3.2 Using a Separate Tip Characterizer

Usually the unknown specimen is chosen for its scientific or technological interest. If we use it to estimate the tip with which it is imaged, we are stuck with its properties, however undesirable they may be for that purpose. For this reason, though it is sometimes possible to use a specimen as its own tip characterizer as in the last section, the more desirable approach will usually be to have a separate characterizer. This separate characterizer should ideally be chosen to have features sharper and as tall as those of interest on the unknown specimen. One advantage of blind reconstruction is that these desirable properties need not be combined in the same feature [21]. That is, it is not necessary for the characterizer to contain high aspect ratio features like tall sharp spikes. For most purposes, tall broad features, provided their edges are sharp, combined with shorter narrower objects work just as well. For unknowns with more than a few tens of nanometers of relief, some combination of the proposed [37, 38] lithographically patterned sharp-edged or undercut objects along with some deliberate surface roughness or deposition of small particles is likely to be effective.

**Fig. 14:**
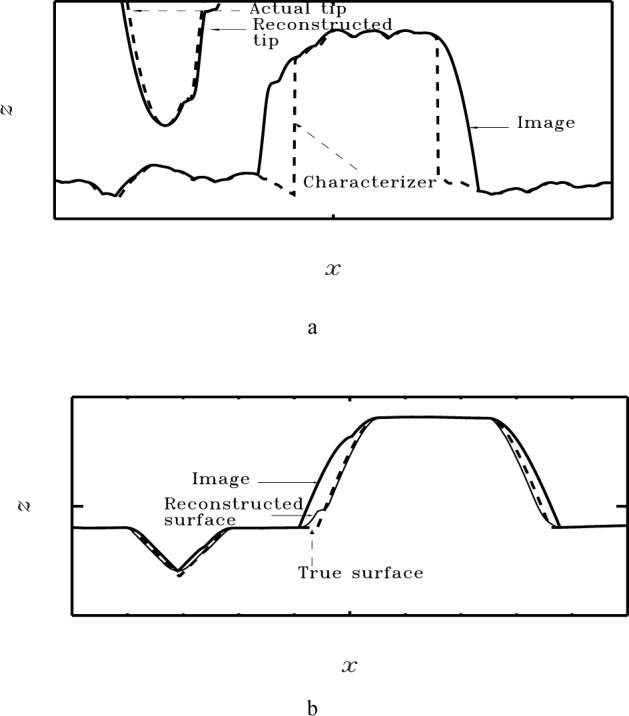
Reconstruction of a specimen surface using blind tip estimation and a separate tip characterizer. (a) The tip is reconstructed from an image of a characterizer with some features sharper than those of the unknown. (b) The unknown is reconstructed from its image.

The process of reconstructing the unknown’s topography is illustrated in Fig. 14. Figure 14a shows reconstruction of the tip shape from the image of a characterizer which has a tall feature and some significant surface roughness. The tip is estimated by using the itip_estimate() routine with the characterizer image as input. The resulting tip is then used to reconstruct the unknown from its image in Fig. 14b by using the ierosion() routine with the unknown’s image and the tip as just estimated. The recommended procedure is to image the characterizer, then the unknown, then the characterizer again. In this way one can obtain before and after estimates of the tip to rule out tip changes during imaging.

Even when using a surface reconstruction technique, it is important to choose tips sharp enough to touch features of interest. It is not possible for any technique to reconstruct what the tip did not touch. Given that the tip is chosen sharp enough, there are two ways to view the results of the above exercise. One can adopt a “rough and ready” philosophy in which one chooses the best tip characterizer possible, then regards the reconstructed surface as the best possible estimate of the true surface. A problem with this approach is the lack of an uncertainty estimate. However, there is a more rigorous approach. Since the reconstructed tip is, if anything, too blunt, the reconstructed surface is an inner bound on the parts of the object touched by the tip [18]. The image is an outer bound. The part of the unknown accessible to the tip is therefore bounded above and below by these two surfaces. Using sharp tips and sharp characterizers narrows the difference between the bounds.

#### 7.3.3 Finding the Largest Tip Consistent With Independent Images

The blind reconstruction algorithm determines the largest tip consistent with all the features within a given image. Sometimes it is useful to calculate the largest tip consistent with *several* images. For example, one might have an edge artifact which is sharp along one direction but translationally symmetric along the orthogonal one. Such an artifact provides good tip characterization along only one axis. However, if several images are acquired with the artifact in different orientations, one has good information along several axes. How best to combine all this information?

**Fig. 15:**
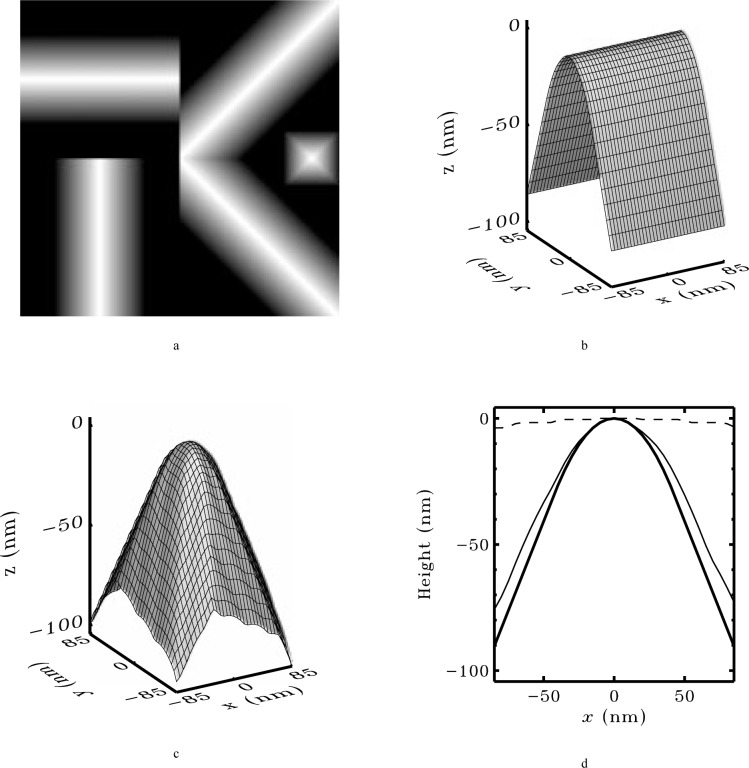
Tip reconstruction consistent with multiple images. (a) Top view of four possible orientations of an edge artifact. At the middle right is a view of the pyramidal tip, showing its orientation with respect to the edge artifacts. (b) Tip reconstructed by imaging a single orientation of the edge. (c) Tip reconstructed using images of the edge in all orientations shown. (d) Comparison of cross sections along the *x* direction through the actual tip (thick line), the tip in (c) (thin line), and the tip in (b) (dashed line). A realistic scale in these simulations was determined by choosing models for tip and edge with end radii (*r*_tip_ = 30 nm, *r*_edge_ = 5 nm) and included angles (*θ*_tip_ = 70.5°, *θ*_edge_ = 70.5° asymptotically far from the edge, less near the edge) consistent with commercial claims for similar objects.

This is straightforward. One simply uses the output of the calculation with one image as the starting estimate for the next one. That is, one calculates itip_estimate(im1,…,tip,…), then passes that value of tip to the routine again in itip_estimate(im2,…,tip,…), and so on through all the available images. Then one cycles through the images again, starting again with im1 until there is no further change in the tip estimate. The advantage of this procedure is illustrated in Fig. 15, where we determine a tip shape from several orientations of an edge artifact. When using multiple images rather than a single one, one must be more concerned about whether the tips which produced the two images were really the same. Was there tip damage when the tip was retracted from the first surface or approached the second? Was the angle of the tip to the surface the same in both instances? If the tips were not actually the same to within the threshold parameter, forcing consistency in this way leads to an incorrect result.

#### 7.3.4 Combining Blind Reconstruction With the Erosion Method

We have discussed two methods for estimating a tip using the image of a tip characterizer. The erosion method, *P*_r_ = *I* ⊖ *S*, requires the geometry of the characterizer to be independently known. Blind reconstruction [Eq. (14) and Eq. (15)] does not. If *S* is known with low enough uncertainty, then the erosion method yields the better result. However, accurate enough knowledge of *S* is difficult to obtain in practice, particularly for characterizers with relief on the scale of hundreds of nanometers. Such tall characterizers are necessary for estimating tip shapes for tall specimens like lithographically patterned semiconductors. At smaller size scales the task of obtaining a known characterizer, while not demonstrated at present, may be more amenable to solution. Molecules (e.g., fullerenes) have consistent shapes determined by bonding interactions. Somewhat larger particles (perhaps colloidal gold [39]) may exist in a size regime where surface tension dominates over bulk forces, so they may adopt a spherical shape. These small particles of known shape, however, only permit characterization of the tip for a distance from the apex less than the particle height.

We therefore anticipate the possibility of mixed characterizers, in which some parts have known shape and other parts do not. For instance, one might decorate the surface of the tall funnel-like structures of Edenfeld et al. [38] with colloidal gold spheres.

**Fig. 16:**
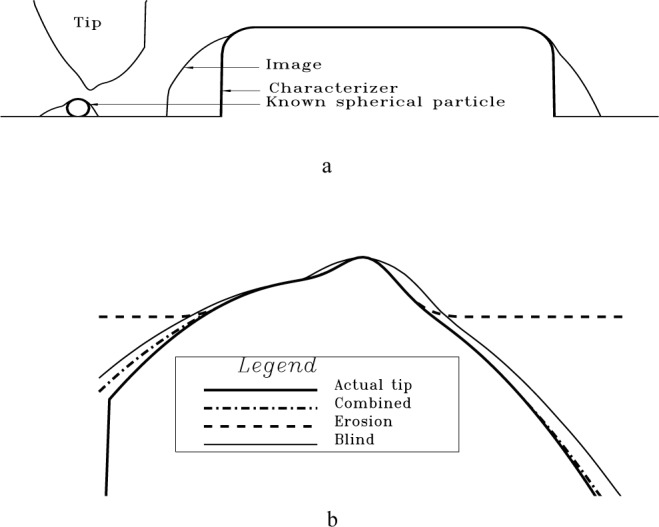
Partially blind reconstruction of a tip. (a) A tip characterizer, tip and calculated image. The tip characterizer is mostly unknown, with the exception of the small spherical particle at left, which is assumed to be of known radius. (b) Comparison (expanded view) of various reconstructed tips with the actual one used in the calculation.

It is useful in this instance to use partially blind reconstruction, as first briefly described in Ref. [21]. The concept and the procedure are explained with the aid of Fig. 16. In Fig. 16a we have a characterizer in which we assume the small spherical particle at the left is known to have radius, *r*. We take from the image the subset which is the image of the sphere, and erode from it a sphere of this size. This is the known-characterizer, or erosion, method of tip estimation [Eq. (11)], and it produces the tip shown as the dashed line in Fig. 16b. This is an outer bound on the tip shape, and hence a valid starting point for a blind reconstruction calculation using the whole image. The result is shown in Fig. 16b as the combined (dash-dot) curve. This is contrasted with completely blind reconstruction (thin continuous line) in which no assumption is made concerning the particle’s size or shape.

In this way we produce a general method for which information about the characterizer is not required, but may be used when available. As is evident in Fig. 16, the erosion method provides good characterization near the tip apex, but no characterization whatever more than a known-particle diameter away. The blind reconstruction result is not as limited in vertical range, but is everywhere too broad by an amount on the order of the particle size. The combination provides the best of both worlds.

## 8. Summary

Distortion of image features due to the non-ideal geometry of tips is an important issue for nanometer-scale metrology, particularly for specimen features with aspect ratios comparable to that of the tip such as are frequently encountered in the study of granular materials, microroughness, lithographically patterned semiconductor electronics, and biological materials.

We have discussed how the tip-sample interaction can be modeled, how and to what extent image distortions may be corrected, and how tip geometries may be estimated from images of tip characterizers. When the characterizer geometry is not independently known, the tip estimation method is called blind reconstruction. The appendices provide algorithms necessary for image simulation, surface reconstruction, and tip estimation. In Secs. 3 through 5 we discussed the basis and some of the details of these algorithms. In Sec. 6 we discussed some of the limitations of the algorithms and introduced a method to stabilize blind reconstruction in the presence of noise. Finally, Sec. 7 provided some practical guidance on the use of the algorithms.

## 9. Appendix A. General Notes Concerning Algorithms

The appendices comprise an annotated listing of a morphology library. That is, if the line numbers, comments and section headers are stripped, what remains should compile on a standard C compiler. *Throughout, comments are in standard typeface, while C code is boldface.* At the time of this writing, the code is available in machine-readable format for anonymous FTP from NIST.^1^ These algorithms are intended to be illustrative implementations of the operations discussed in the main body of the text. Since some of the algorithms reported here are newly derived, their derivation and correctness have been the principal concerns of this study. Efficiency has been a lesser concern, and although some steps to improve execution time are included no representation is made here that these routines are optimum in that respect.

The routines as presented were all formulated to use integer arithmetic. There are two reasons for this. First, commercial instruments read images using analog to digital converters. The data are therefore typically 12 or 16 bit integers. Second, the certainty map and tip estimation algorithms employ tests of equality which are relatively simple when integer arithmetic is used, since there is then no problem with round-off error.

In order to conveniently employ two dimensional arrays of variable dimension, such as those required to represent images, surfaces, and tips, we use pointers of type long **. Utility routines, allocmatrix() and freematrix(), are provided in Appendix B for creating and freeing arrays addressed this way. Appendix B also contains a reflection routine, ireflect (T, m, n), which is provided as a utility. It reflects the array T (size *m* × *n*) through the origin. Since all the morphology and tip estimation routines provided here require (or produce) reflected versions of the tip, the ireflect() function may be useful either to place the tip in that form or to convert a result back to the real-space geometry.

If it is necessary to perform these operations on floating point or double precision arrays, there are two possibilities. The routines below may be changed to accept a different data type by changing the appropriate type declaration statements. The programmer who does this should be aware that some of the algorithms employ tests of equality which will need to be modified for floating or double precision data types due to the possibility of round-off error. Alternatively the arrays can be appropriately scaled, converted to integers, the morphology operations performed, converted back to floating point and rescaled. For example, to compute *C* = *A* ⊕ *B*, one computes *C* = (*αA* ⊕ *αB*)/*α*. The scale factor, *α*, is any convenient factor chosen to scale *A* and *B* to a useful range of integer values for the dilation operation. For example, if *A* and *B* are floating point arrays taking on values ranging from 0 to 1, one might choose *α* = 10^5^, since this should provide ample resolution while remaining safely within the range of 4 byte long integers.

## 10. Appendix B. Header Material and Utility Routines

### 10.1 Header

Include files

1 #[include <stdio.h>
2 #[include <memory.h>

Some useful Macros

3 #[define MIN(a,b) ((a)<(b)?a:b)
4 #[define MAX(a,b) ((a)>(b)?a:b)

Function declarations

5 long **ireflect();
6 long **idilation();
7 long **ierosion();
8 long **icmap();
9 long **iopen();
10 long **allocmatrix();
11 long itip_estimate_iter();
12 long itip_estimate_point();
13 void itip_estimate();
14 void itip_estimate0();
15 long useit();
16 void freematrix();

### 10.2 Array Allocation and De-allocation

The following routines allow allocation and freeing of matrices

17 long **allocmatrix(ysiz,xsiz)
18 long ysiz,xsiz;

Allocates a long integer matrix of dimension [ysiz][xsiz] using an array of pointers to rows. ysiz is the number of rows. xsiz is the number of columns.

19 {
20  long **mptr, points to allocated matrix i; counter

Allocate pointers to rows

21 Smptr = (long **)malloc(ysiz*sizeof(long*));
22  if (mptr == NULL) {
23   printf(“Error: Allocation of mptr failed”);
24   printf(“ in allocmatrix\n”);
25   exit(1);
26  }

Allocate rows

27  for (i=0;i<ysiz;i++) {
28   mptr[i]=(long *)malloc(xsiz*sizeof(long));
29   if (mptr[i] == NULL) {
30    printf(
31      “Error: Allocation of mptr[%ld]”,i);
32    printf(“ failed in allocmatrix\n”);
33    exit(1);
34   }
35  }

Done. Return result.

36  return mptr;
37 }
38 void freematrix(mptr,ysiz)

Frees memory allocated with allocmatrix

39  long **mptr;
40  long ysiz;
41  {
42   long i;
43   for (i=0;i<ysiz;i++) free(mptr[i]);
44   free(mptr);
45  }

### 10.3 Reflection

The following routine performs reflection of integer arrays.

46  long **ireflect(surface,surf_xsiz,surf_ysiz)
47  long **surface, surface is a surf_ysiz 3 surf_xsiz array surf_xsiz,surf_ysiz; size of surface
48  {
49   long **result;
50   long i,j; index

Create output array of appropriate size

51  result = allocmatrix(surf_ysiz,surf_xsiz)
52  for (j=0;j<surf_ysiz;j++) { Loop over all points in output array
53    for (i=0;i<surf_xsiz;i++) {
54     result[j][i] =2surface[surf_ysiz-1-j][surf_xsiz-1-i];
55    }
56   }
57   return(result);
58  }

## 11. Appendix C. Elementary Morphology Routines

### 11.1 Dilation

The following routine performs dilation on integer arrays.

59 long **idilation(surface,surf_xsiz, surf_ysiz, tip,tip_xsiz,tip_ysiz,xc,yc)
60 long **surface, surface is a surf_ysiz × surf_xsiz array surf_xsiz,surf_ysiz, size of surface **tip, tip is a tip_ysiz × tip_xsiz array representing the *reflected* tip (i.e., *p*(*x*,*y*) in Eq. (3)) tip_xsiz,tip_ysiz, size of tip xc,yc; center coordinates of tip
61 {
62  long **result;
63  long i,j,px,py; index
64  long max;
65  long pxmin,pxmax,pymin,pymax; range of indices into tip
66  long temp;

Create output array of appropriate size.

67  result = allocmatrix(surf_ysiz,surf_xsiz);
68  for (j=0;j<surf_ysiz;j++) { Loop over all points in output array

Compute allowed range of py. This may be different from the full range of the tip due to edge overlaps.

69  pymin = MAX(j-surf_ysiz+1,-yc);
70  pymax = MIN(tip_ysiz-yc-1,j);
71  for (i=0;i<surf_xsiz;i++) {

Compute allowed range of px. This may be different from the full range of the tip due to edge overlaps.

72  pxmin = MAX(i-surf_xsiz+1,-xc);
73  pxmax = MIN(tip_xsiz-xc-1,i);
74  max = surface[j-pymin][i-pxmin] + tip[pymin+yc] [pxmin+xc];
75  for (px=pxmin;px<=pxmax;px++) { Loop over points in tip
76   for (py=pymin;py<=pymax;py++) {

The next line calculates the term in square brackets in Eq. (4). For comparison purposes, note that i, j, px, and py here play the roles of *x*, *y*, *u*, and *v* respectively. The xc, yc offsets allow the point (px,py)=(0,0) to reference the tip “center” (usually the apex) placed somewhere in the interior of the tip array.

77       temp = surface[j-py][i-px] + tip[py+yc][px+xc];
78       max = MAX(temp,max);
79      }
80     }
81     result[j][i] = max;
82    }
83   }
84   return(result);
85  }

### 11.2 Erosion

The following routine performs erosion on integer arrays.

86 long **ierosion(image,im_xsiz,im_ysiz,tip, tip_xsiz,tip_ysiz,xc,yc)
87 long **image, image is an im_ysiz × im_xsiz array im_xsiz,im_ysiz,size of image **tip, tip is a tip_ysiz × tip_xsiz array representing the *reflected* tip (i.e., *p*(*x*,*y*) in Eq. (3)) tip_xsiz,tip_ysiz, size of tip xc, yc; center coordinates of tip
88 {
89  long **result;
90  long i,j,px,py; index
91  long min;
92  long pxmin,pxmax,pymin,pymax; range of indices into tip
93  long temp;

Create output array of appropriate size

94  result = allocmatrix(im_ysiz,im_xsiz);
95  for (j=0;j<im_ysiz;j++) { Loop over all points in output array

Compute allowed range of py. This may be different from the full range of the tip due to edge overlaps.

96  pymin = MAX(-j,-yc);
97  pymax = MIN(tip_ysiz-yc,im_ysiz-j)-1;
98  for (i=0;i<im_xsiz;i++) {

Compute allowed range of px. This may be different from the full range of the tip due to edge overlaps.

99  pxmin = MAX(-xc,-i);
100  pxmax = MIN(tip_xsiz-xc,im_xsiz-i)-1;
101  min = image[j+pymin][i+pxmin]-tip[pymin+yc][pxmin+xc];
102  for (px=pxmin;px<=pxmax;px++) { Loop over points in tip
103   for (py=pymin;py<=pymax;py++) {

The next line calculates the term in square brackets in Eq. (12). For comparison purposes, note that i, j, px, and py here play the roles of *x*, *y*, *u*, and *v* respectively. The xc, yc offsets allow the point (px, py) = (0,0) to reference the tip “center” (usually the apex) placed somewhere in the interior of the tip array.

104       temp = image[j+py][i+px]2 tip[py+yc][px+xc];
105       min = MIN(temp,min);
106      }
107     }
108     result[j][i] = min;
109    }
110   }
111   return(result);
112  }

### 11.3 Opening

The opening of *A* by *B* is denoted *A* ° *B* and defined as *A* ° *B* = (*A* ⊖ *B*) ⊕ *B*. This routine is used by the tip estimation routines to save time, since points where *I* ° *P* = *I* do not produce tip refinement and need not be considered.

113 long **iopen(image,im_xsiz,im_ysiz, tip,tip_xsiz,tip_ysiz)
114 long **image, image is an im_xsiz3im_ysiz array im_xsiz,im_ysiz, size of image **tip, tip is a tip_ysiz3tip_xsiz array tip_xsiz,tip_ysiz; size of tip
115 {
116 long **result,**eros;
117 eros = ierosion(image,im_xsiz,im_ysiz, tip,tip_xsiz,tip_ysiz,tip_xsiz/2, tip_ysiz/2);
118 result = idilation(eros,im_xsiz,im_ysiz, tip,tip_xsiz,tip_ysiz,tip_xsiz/2, tip_ysiz/2);
119 freematrix(eros,im_ysiz); free intermediate result
120 return(result);
121 }

## 12. Appendix D. Certainty Map

122 long **icmap(image,im_xsiz,im_ysiz,tip, tip_xsiz,tip_ysiz,rsurf,xc,yc)
123 long **image, image is an im_ysiz3im_xsiz array im_xsiz,im_ysiz, size of image **tip, tip is a tip_ysiz3tip_xsiz array tip_xsiz,tip_ysiz, size of tip **rsurf, rsurf is an im_ysiz im_xsiz array xc,yc; center coordinates of tip
124 {
125 long **cmap;
126 long imx,imy,tpx,tpy; index
127 long tpxmin,tpxmax,tpymin,tpymax;
128 long count;
129 long rxc,ryc; center coordinates of reflected tip
130 long x,y;
131 rxc = tip_xsiz-1-xc; compute center of unreflected tip
132 ryc = tip_ysiz-1-yc;

Create output array of appropriate size. Initialize all entries to 0.

133 cmap = allocmatrix(im_ysiz,im_xsiz);
134 for (imy=0;imy<im_ysiz;imy++)
135 for (imx=0;imx<im_xsiz;imx++) cmap[imy][imx]=0;

Loop over all pixels in the interior of the image. We skip pixels near the edge. Since it is possible there are unseen touches over the edge, we must conservatively leave these cmap entries at 0.

136  for (imy=ryc;imy<=im_ysiz+ryc-tip_ysiz;imy++) {
137   for (imx=rxc;imx<=im_xsiz+rxc-tip_xsiz;imx++) {
138    tpxmin = MAX(0,rxc-imx);
139    tpxmax = MIN(tip_xsiz-1,im_xsiz-1 +rxc-imx);
140    tpymin = MAX(0,ryc-imy);
141    tpymax = MIN(tip_ysiz-1,im_ysiz-1 +ryc-imy);
142    count = 0;
143    for (tpy=tpymin;tpy<=tpymax&&count<2; tpy++) {
144     for (tpx=tpxmin; tpx<=tpxmax&&count<2;tpx++) {
145      if (image[imy][imx]-tip[tip_ysiz-1-tpy][tip_xsiz-1-tpx] == rsurf[tpy+imy-ryc][tpx+imx-rxc]) {
146       count++; increment count
147       x = tpx+imx-rxc; remember coordinates
148       y = tpy+imy-ryc;
149      }
150     }
151    }
152    if (count==1) cmap[y][x] = 1; single contact = good reconstruction
153   }
154  }
155  return(cmap);
156  }

## 13. Appendix E. Blind Tip Estimation Routines

### 13.1 Tip Estimate From a Single Image Point

The following routine is a basic building block from which the more complete tip estimation routines to follow are constructed. It computes the tip shape as deduced from a single i,j image coordinate.

The threshold parameter is used as follows: A new estimate of the tip height at a given pixel is computed according to the formula given by Eq. (14). This value is then augmented by thresh. If the old estimate is less than the augmented new estimate, no action is taken. Otherwise, the value is changed to agree with the augmented new estimate. Thus, if thresh = 0, this routine implements Eq. (14) directly. Larger values of threshold give greater noise immunity at the cost of degrading the estimate.

The interior of the image is easier (and safer) to use in calculating the tip estimate. At the edges part of the tip extends beyond the edge where the image values are unknown. It is possible to handle this case by making worst case assumptions and thereby extract some information even from the data near the edge. Below, I separate the code into two parts, one which handles the interior and one which handles the edges. This serves two functions. First, because the edges can be tricky to handle properly, I had more than the usual share of errors in this part of the code during development. It was useful to be able to turn this part of the code on or off at will. Second: The execution of the routine is slightly faster with the code divided since it is not then necessary to do checking for special cases in the part of the code that handles the interior.

To compile code which skips the edges, set USE_EDGES to 0 on the next line.

157 #[**define USE_EDGES 1** set to 1 to include edges, 0 to exclude
158 #[**define MINUS_INF** 2**2147483648L** smallest 4 byte integer
159 #[**define INFINITY 2147483647L** largest 4 byte integer
160 **long itip_estimate_point(ixp,jxp,image, im_xsiz,im_ysiz,tip_xsiz,tip_ysiz,xc,yc, tip0,thresh)**
161 **long ixp,jxp**, image coordinate, x′ = (ixp,jxp), for which the calculation is performed

****image**, image array

**im_xsiz,im_ysiz**, x,y size of image array

**tip_xsiz,tip_ysiz**, x,y size of tip to be determined

**xc,yc**, center coordinates of tip

****tip0**, starting est. Size must be tip_xsiz by tip_ysiz

**thresh;** threshold value to use

162 **{**
163 **long ix,jx**, index into the output tip array (x) **id,jd;** index into p′ (d)
164 **long temp,imagep,dil;** various intermediate results
165 **long count=0;** counts places where tip estimate is improved
166 **long interior;**
167 **int apexstate**, States and their possible values **xstate**,

**inside = 1**, Point is inside image

**outside = 0;** Point is outside image

168 **interior = jxp>=tip_ysiz-1 && jxp<=im_ysiz-tip_ysiz && ixp>=tip_xsiz-1 && ixp<=im_xsiz-tip_xsiz;**

First handle the large middle area where we don’t have to be concerned with edge problems. Because edges are far away, we can leave out the overhead of checking for them in this section.

169 **if (interior) {**
170  **for (jx=0;jx<tip_ysiz;jx++) {**
171   **for (ix=0;ix<tip_xsiz;ix++) {**
172    **imagep = image[jxp][ixp];**
173    **dil = MINUS_INF;** initialize maximum to − ∞
174    **for (jd=0;jd<tip_ysiz;jd++) {**
175     **for (id=0;id<tip_xsiz;id++) {**

The next line implements the condition contained in Eq. (19). Here ixp, jxp, id, jd, xc, and yc take the place of *x*′, *y*′, *d_x_*, *d_y_*, *x_c_* and *y_c_*. The following one evaluates the term in square brackets in Eq. (17).

176      **if (imagep-image[jxp+yc-jd] [ixp +xc-id] > tip0[jd][id]) continue;**
177      **temp = image[jx+jxp-jd][ix+ixp-id]+ tip0[jd][id]-imagep;**
178      **dil = MAX(dil,temp);**
179     **}** end for id
180    **}** end for jd
181    **if (dil == MINUS_INF) continue;**
182    **tip0[jx][ix] = dil<tip0[jx][ix]-thresh?(count++,dil+thresh): tip0[jx][ix];**
183    **}** end for ix
184   **}** end for jx
185   **return(count);**
186  **}** endif
187  #[**if USE_EDGES**

Now handle the edges

188 **for (jx=0;jx<tip_ysiz;jx++) {**
189  **for (ix=0;ix<tip_xsiz;ix++) {**
190   **imagep = image[jxp][ixp];**
191   **dil = MINUS_INF;** initialize maximum to −∞
192    **for (jd=0;jd<=tip_ysiz-1 && dil<INFINITY;jd++) {**
193    **for (id=0;id<=tip_xsiz-1;id++) {**

Determine whether the tip apex at (xc,yc) lies within the domain of the translated image, and if so, if it is inside (i.e., below or on the surface of) the image.

194   **apexstate = outside;** initialize
195   **if (jxp+yc-jd<0** ║ **jxp+yc-jd>= im_ysiz** ║ **ixp+xc-id<0** ║ **ixp+xc-id>= im_xsiz) apexstate = inside;**
196   **else if (imagep-image[jxp+yc-jd][ixp+xc-id] <=tip0[jd][id]) apexstate = inside;**

Determine whether the point (ix, jx) under consideration lies within the domain of the translated image.

197   **if (jxp+jx-jd<0** ║ **jxp+jx-jd>=im_ysiz** ║ **ixp+ix-id<0** ║ **ixp+ix-id>= im_xsiz) xstate = outside;**
198   **else xstate = inside;**

There are 3 actions we might take, depending upon which of 4 states (2 apexstate possibilities × 2 xstate ones) we are in.

If apexstate == outside, the condition in Eq. (19) is not satisfied. Regardless of xstate, no change is made for this (id,jd).

199 **if (apexstate==outside) continue;**

If apex is inside and x is outside worst case is image[jx+jxp-jd] [ix+ixp-id] →∞. This would result in no change for *any* (id,jd). We therefore abort the loop and go to next (ix,jx) value.

200 **if (xstate==outside) goto nextx;**

The only remaining possibility is x and apex both inside. This is the same case we treated in the interior.

201    **temp = image[jx+jxp-jd][ix+ixp-id]+tip0[jd][id]-imagep;**
202    **dil = MAX(dil,temp);**
203   **}** end for id
204  **}** end for jd
205  **if (dil == MINUS_INF) continue**;
206  **tip0[jx][ix] = dil<tip0[jx][ix]-thresh?(count++,dil+thresh): tip0[jx][ix];**
207 **nextx:;**
208 **}** end for ix
209 **}** end for jx
210 **return(count);**
211 [**endif**
212 **}**

### 13.2 Full Tip Estimation Algorithm

The following routine estimates tip size by calling tip_estimate_iter until it converges.

213 **void itip_estimate (image,im_xsiz,im_ysiz, tip_xsiz,tip_ysiz,xc,yc,tip0,thresh)**
214 **long **image**, image array

**im_xsiz,im_ysiz**, x,y size of image array

**tip_xsiz,tip_ysiz**, x,y, size of tip to be determined

**xc,yc**, center coordinates of the tip

****tip0**, starting estimate. Must be tip_xsiz by tip_ysiz in size

**thresh;** threshold value to use

215 **{**
216 **long iter=0;**
217 **long count=1;**
218 **while (count) {**
219  **iter++;**
220  **count = itip_estimate_iter(image,im_xsiz, im_ysiz,tip_xsiz,tip_ysiz,xc,yc,tip0, thresh);**
221  **printf (“Finished iteration** [**%ld. “,iter);**
222  **printf (“%ld image locations “,count);**
223  **printf (“produced refinement.\n”);**
224  **}**
225 **}**

The following routine performs one iteration of the tip estimation recursion algorithm through all image pixels. The values of the revised estimate replace those in tip0, and the number of pixels where the value was changed is returned.

226 **long itip_estimate_iter(image,im_xsiz, im_ysiz,tip_xsiz,tip_ysiz,xc,yc,tip0, thresh)**
227 **long **image**, image array

**im_xsiz,im_ysiz**, x,y size of image array

**tip_xsiz, tip_ysiz**, x,y, size of tip to be determined

**xc,yc**, center coordinates of tip

****tip0**, starting est. Size must be tip_xsiz by tip_ysiz

**thresh;** threshold value to use

228 **{**
229 **long ixp,jxp;** index into the image (x′)
230 **long **open;**
231 **long count=0;** counts places where tip estimate is improved
232 **open = iopen(image,im_xsiz,im_ysiz,tip0, tip_xsiz,tip_ysiz);**
233 **for (jxp=tip_ysiz-1-yc;jxp<=im_ysiz-1-yc; jxp++) {**
234  **for (ixp=tip_xsiz-1-xc;ixp<=im_xsiz-1-xc;ixp++) {**
235   **if (image[jxp] [ixp] -open[jxp] [ixp] > thresh) if (itip_estimate_point (ixp, jxp, image,im_xsiz,im_ysiz,tip_xsiz, tip_ysiz,xc, yc,tip0,thresh)) count++;**
236   **}**
237 **}**
238 **freematrix(open,im_ysiz);**
239 **return(count);**
240 **}**

### 13.3 Partial Tip Estimation Algorithm

The tip estimation routine in Sec. 13.2 is complete but may be expensive in compute time. The routine below is a faster partial calculation which iterates itip_estimate_point() through a relatively small number of image points chosen because they are among those that generate the most refinement of the tip shape. The result may be useful in either of two ways. It may be used as the tip estimate by itself when a cruder estimate is sufficient.

Alternatively, it may be used as a starting estimate for the full algorithm in Sec. 13.2. Using itip_estimate0() prior to calling itip_estimate() can save execution time compared to calling itip_estimate() directly. This is because the order of evaluation of the points can affect the execution speed. The image at some locations (e.g., isolated local maxima) puts great constraints on the tip shape. If the tip shape is refined by considering these points first, time is saved later. The itip_estimate_point() routine in Sec. 13.1, since it performs the calculation at a single point, allows the user to select the order in which image coordinates are considered. In using the routine in this mode, the fact that the refined tip replaces tip0 means results of one step automatically become the starting point for the next step. The routine returns a long integer which is the number of pixels within the starting tip estimate which were updated.

241 void itip_estimate0(image,im_xsiz,im_ysiz, tip_xsiz,tip_ysiz,xc,yc,tip0,thresh)
242 long **image, image array

im_xsiz,im_ysiz, x,y size of image array

tip_xsiz,tip_ysiz, x,y, size of tip to be determined

xc,yc, center coordinates of the tip

**tip0, starting estimate. Must be tip_xsiz by tip_ysiz in size

thresh; threshold value to use

243 {
244  long i,j,n;
245  long arraysize; size of array allocated to store list of image maxima
246  long *x,*y; point to coordinates of image maxima
247  long iter=0;
248  long count;
249  long delta; defines what is meant by near neighborhood for purposes of point selection.

We need to create temporary arrays to hold a list of selected image coordinates. The space needed depends upon the image. This creates a memory management issue. We address it by allowing for 300 maxima—a good number (corresponding perhaps to a grainy surface) which should be big enough for most images. Then we monitor memory usage and reallocate a larger array if necessary.

Coordinates to be used are determined by the routine useit, below, which returns either 1 (if the given coordinate is to be used) or 0 (if not). The user can substitute his own version of this routine if he believes he has a more economical algorithm for choosing points.

250 arraysize = 300;
251 x = (long *)malloc(arraysize*sizeof(long));
252 if (x == NULL) {
253  printf(“Unable to allocate x array in”);
254  printf(“ itip_estimate0 routine.\n”);
255  return;
256 }
257 y = (long *)malloc(arraysize*sizeof(long));
258 if (y == NULL) {
259  printf(“Unable to allocate y array in”);
260  printf(“ itip_estimate0 routine.\n”);
261  free(x);
262  return;
263 }

Now choose a nearest neighborhood size to send the useit routine. The neighborhood should be at least equal to 1 (i.e., consider all points with x,y within ±1 of the one under consideration). Otherwise ALL points are used, equivalent to the full itip_estimate routine, and no speed advantage is derived, which loses the whole point of having a tip_estimate0. However, the size of the neighborhood should in principle scale with the size of the tip. I use a small fraction of tip size (1/10) because in practice the routine seems to run acceptably quickly even at this setting—there’s no point in sacrificing performance for speed if the present speed is acceptable.

264 delta = MAX(MAX(tip_xsiz,tip_ysiz)/10,1);

Create a list of coordinates to use.

265 n=0; Number of image maxima found so far
266 for (j=tip_ysiz-1-yc;j<=im_ysiz-1-yc; j++) {
267  for (i=tip_xsiz-1-xc;i<=im_xsiz-1-xc; i++){
268   if (useit(i,j,image,im_xsiz,im_ysiz, delta)) {
269    if (n == arraysize) { need more room in temporary arrays
270     arraysize *= 2; increase array size by factor of 2
271     x = (long *)realloc(x, arraysize*sizeof(long));
272     if (x == NULL) {
273      printf(“Unable to realloc x”);
274      printf(“array in itip_”);
275      printf(“estimate0.\n”);
276      free(y);
277      return;
278     }
279     y = (long *)realloc(y, arraysize*sizeof(long));
280     if (y == NULL) {
281      printf(“Unable to realloc y”);
282      printf(“array in itip_”);
283      printf(“estimate0.\n”);
284      free(x);
285      return;
286     }
287    }
288    x[n] = i; We found another one
289    y[n] = j;
290    n++;
291   }
292   }
293 }
294 printf(“Found %ld internal local maxima\n”,n);

Now refine tip at these coordinates recursively until no more change

295 do {
296  count = 0;
297  iter++;
298  for (i=0;i<n;i++)
299   if (itip_estimate_point(x[i],y[i], image,im_xsiz,im_ysiz,tip_xsiz, tip_ysiz,xc,yc,tip0,thresh)) count++;
300  printf(“Finished iteration [%ld.”,iter);
301  printf(“ %ld image locations “,count);
302  printf(“produced refinement.\n”);
303 }while (count);

Free temporary space

304 free(x); free(y);
305 }

The following is a routine that determines whether a selected point at coordinates x, y within an image is deemed to be suitable for image refinement. In this implementation, the algorithm simply decides to use the point if it is a local maximum of the image. It defines a local maximum as a point with height greater than any of its near neighbors.

306 long useit(x,y,image,sx,sy,delta)
307 long x,y; Coordinates of selected point within image
308 long **image; pointer to image
309 long sx,sy; x and y size of image
310 long delta; size of neighborhood to search for maximum. Searches interval ([x-delta,x+delta],[y-delta,y+delta]).
311 {
312 long xmin,xmax,ymin,ymax; actual interval to search
313 long i,j;
314 long max=image[y][x]; value of maximum height in the neighborhood
315 xmin = MAX(x-delta,0);
316 xmax = MIN(x+delta,sx-1);
317 ymin = MAX(y-delta,0);
318 ymax = MIN(y+delta,sy-1);
319 for (j=ymin;j<=ymax;j++) {
320 for (i=xmin;i<=xmax;i++) {
321 max = image[j][i]>max?image[j][i]:max;
322  }
323 }
324 if (max == image[y][x]) return(1);
325 return(0);
326 }
